# Spontaneous Proliferation of CD4^+^ T Cells in RAG-Deficient Hosts Promotes Antigen-Independent but IL-2-Dependent Strong Proliferative Response of Naïve CD8^+^ T Cells

**DOI:** 10.3389/fimmu.2018.01907

**Published:** 2018-08-23

**Authors:** Juhee Kim, Jun Young Lee, Kyungjin Cho, Sung-Wook Hong, Kwang Soon Kim, Jonathan Sprent, Sin-Hyeog Im, Charles D. Surh, Jae-Ho Cho

**Affiliations:** ^1^Academy of Immunology and Microbiology, Institute for Basic Science, Pohang, South Korea; ^2^Department of Integrative Biosciences and Biotechnology, Pohang University of Science and Technology, Pohang, South Korea; ^3^Immunology Division, Garvan Institute of Medical Research, Darlinghurst, NSW, Australia; ^4^University of New South Wales, Sydney, NSW, Australia

**Keywords:** spontaneous proliferation, lymphopenia-induced homeostatic proliferation, naïve CD4 T^+^ cells, naïve CD8^+^ T cells, interleukin-2 (IL-2), specific pathogen-free (SPF), germ-free (GF)

## Abstract

The fast and intense proliferative responses have been well documented for naïve T cells adoptively transferred into chronic lymphopenic hosts. This response known as spontaneous proliferation (SP), unlike antigen-independent lymphopenia-induced proliferation (LIP), is driven in a manner dependent on antigens derived from commensal microbiota. However, the precise nature of the SP response and its impact on homeostasis and function for T cells rapidly responding under this lymphopenic condition are still unclear. Here we demonstrate that, when naïve T cells were adoptively transferred into specific pathogen-free (SPF) but not germ-free (GF) RAG^−/−^ hosts, the SP response of these cells substantially affects the intensity and tempo of the responding T cells undergoing LIP. Therefore, the resulting response of these cells in SPF RAG^−/−^ hosts was faster and stronger than the typical LIP response observed in irradiated B6 hosts. Although the intensity and tempo of such augmented LIP in SPF RAG^−/−^ hosts were analogous to those of antigen-dependent SP, the former was independent of antigenic stimulation but most importantly, dependent on IL-2. Similar observations were also apparent in other acute lymphopenic settings where antigen-dependent T cell activation can strongly occur and induce sufficient levels of IL-2 production. Consequently, the resulting T cells undergoing IL-2-driven strong proliferative responses showed the ability to differentiate into functional effector and memory cells that can control infectious pathogens. These findings therefore reveal previously unappreciated role of IL-2 in driving the intense form of T cell proliferative responses in chronic lymphopenic hosts.

## Introduction

Proliferation of naïve T cells under lymphopenic environments has long been accepted as a crucial homeostatic mechanism by which a diverse repertoire of these cells can be stably maintained at constant number during their peripheral life-time (1, 2). Therefore, this proliferative response, also known as lymphopenia-induced homeostatic proliferation (LIP), is considered as a common phenomenon for the most polyclonal and even monoclonal naïve T cell repertoire adoptively transferred into a recipient animal under various lympho-depletion settings either genetically (e.g., RAG^−/−^, TCRβ^−/−^, CD3ε^−/−^, and SCID mice) or conditionally (e.g., mice treated with irradiation and cytotoxic agents (3–7). The LIP response in these hosts is relatively slow with 2–3 rounds of cell division per week and is driven by a signal from self-ligands and elevated levels of IL-7 (3, 7–10). Despite the slow proliferation is unique for the LIP response in lymphopenic hosts, the relative strength of this response can be modulated variably depending on the cytokines being engaged (11). For instance, the LIP response driven by IL-2 and/or IL-15 is far stronger than those induced by IL-7 (12–14).

In sharp contrast to the LIP, however, totally different form of proliferative responses has also been reported for naïve T cells when adoptively transferred into the aforementioned chronic lymphopenic hosts, such as RAG^−/−^ and TCRβ^−/−^ (or CD3ε^−/−^) mice (15–17). The proliferative response in these hosts, known as spontaneous proliferation (SP), is much faster and stronger than those of typical LIP with more than ~7 rounds of cell divisions per week and, unlike LIP, is driven in a manner independent of self-ligands and IL-7 (7, 15). Although the exact nature of the stimuli for the SP response is still incompletely understood, it has been demonstrated that this response is exclusively dependent on largely two key signals, namely from TCR engagements with its cognate foreign-peptide/major histocompatibility complex (foreign-pMHC) ligands and also from costimulatory interactions via CD28 (7, 15). The antigenic stimuli are thought to be derived from commensal microbiota because the SP response of naïve T cells is observed only in RAG^−/−^ hosts raised under the specific pathogen-free (SPF), but not the germ-free (GF), condition (15). Precisely how the commensal antigens are presented to stimulate the SP response of T cells in these hosts and if so, why this phenomenon fails to occur in other lymphopenic hosts, such as irradiated C57BL/6 (B6) mice, remains to be addressed.

In this respect, recent studies have shown that the role of commensal microbial antigens in driving the SP response is not direct but rather indirect effect on T cells via a mechanism dependent on innate immune stimulation through toll-like receptor (TLR) on dendritic cells (DCs) (18–20). Therefore, it is possible that the SP response that occurs in chronic lymphopenic hosts is regulated at least in part, if not exclusively, by some forms of antigen-independent responses other than direct TCR engagements with foreign-pMHC ligands. In fact, it has been shown that IL-6 produced from DCs that are activated by bacterial ligands serves as a major driver for promoting the SP response of naïve T cells adoptively transferred into RAG^−/−^ hosts (18, 21). However, how these data supporting a role of antigen-independent components would be reconciled with the stringent requirement of antigen-dependent components of TCR stimulation for inducing the robust SP response in chronic lymphopenic hosts is largely unclear. In this study, we address these issues by investigating the mechanism of how the SP response of polyclonal or monoclonal T cells is regulated and influences their homeostasis and function during their recovery phase from various settings of lymphopenia. We show here that the SP response of naïve T cells observed in the lymphopenic hosts consists of at least two forms of intensive proliferative responses, namely an antigen-dependent “true” SP response and an antigen-independent but IL-2-dependent SP-like “bystander” response.

## Materials and methods

### Mice

C57BL/6 (B6), B6.PL (Thy1.1), B6.SJL (Ly5.1), Foxp3-eGFP (22), RAG^−/−^, L-2^+/−^ mice, all on a B6 background, were purchased from The Jackson Laboratory. Sources of OT-I.RAG^−/−^, HY and SMARTA TCR Tg mice were previously described (4, 5, 23). Germ free (GF) mice are maintained sterilely at POSTECH Biotech Center (PBC, Korea) as described (24). OT-I.RAG^−/−^.Thy1.1, SMARTA.Thy1.1 and IL-2^−/−^ mice were generated as described (4, 13). Unless it is described, 6–10 weeks old mice were used for the experiments according to the protocols approved by the Animal Experimental and Ethic Committee at the Institute for Basic Science (Korea).

### Naïve T cell purification

Pooled (inguinal, axillary, cervical, and mesenteric) lymph node cells from SMARTA TCR Tg or Foxp3-eGFP mice were prepared for cell sorting as previously described (13, 25), with slight modifications. In brief, LN cells were first depleted of non-T cells by using the following biotinylated antibodies; CD11b, CD11c, CD24, CD19, B220, NK1.1, and IMag according to the manufacturer's protocol (BD biosciences). Enriched T cells were stained with fluorochrome conjugated antibodies to CD8α, CD4, CD44, CD62L, and/or CD5 and then either Foxp3-eGFP^−^ CD4^+^ CD44^lo^ CD62L^+^ (naïve CD4^+^), CD8α^+^ CD62L^+^ CD44 ^lo^ (naïve CD8^+^), CD8α^+^ CD62L^+^ CD44 ^lo^ CD5^hi^ CD4^−^ (naïve CD8^+^ CD5^hi^), and CD8α^+^ CD62L^+^ CD44 ^lo^ CD5^lo^ CD4^−^ (naïve CD8^+^ CD5^lo^) populations were sorted by using a Moflo XDP (Beckman Coulter, Brea, CA, USA) to >95% purity.

### Adoptive transfer

After purification, T cells were labeled with 5 μM of either CFSE (Invitrogen) or CellTrace^TM^ Violet (Molecular Probes), as previously described (26) and injected i.v. into hosts. For inducing lymphopenia, normal B6 mice were treated with anti-Thy1.2 mAb 30-H12 (anti-Thy1.2) (Bio X Cell, i.p. injection in a single dose of 200 μg/mouse, 2 days before cell transfer) or 600cGy of whole-body irradiation (1 day before cell transfer). For generating antigen-induced “SP-like” response, SMARTA CD4^+^ T cells were transferred into the hosts, as indicated in the figures, followed by either LCMV Armstrong (2 × 10^5^ PFU) or LCMV peptide GP_61−80_ (20 μg/mouse) through i.p. injection 1 day post cell transfer. OT-I CD8^+^ T cells were transferred into the hosts, as indicated in the figures, followed by immunization of OVA protein (Sigma Aldrich, 100 μg/mouse). HY.CD8^+^ cells from female HY mice were transferred into female hosts.

### Reconstitution of IL-2^−/−^ T cells

Bone marrow (BM) cells were obtained from B6.SJL (Ly5.1) and IL-2^−/−^ (Thy1.2) mice, mixed at 1:1 ratio. T cell-depletion was done in incubating BM cells with anti-CD4 (RL172), anti-CD8 (3.168), anti-CD24 (J11d) on ice for 10 minutes before adding complement (guinea pig). B6.PL (Thy1.1) mice were lethally irradiated (9.6Gy) before being injected i.v. with 4 × 10^6^ T cell-depleted BM cells. At 8 weeks after BM cell transfer, IL-2^−/−^ naive CD4^+^ T cells (CD90.2^+^ CD44^lo^ CD62L^hi^ CD4^+^) were obtained from these mixed chimeras by FACS sorting.

### Tissue preparation

Single-cell suspensions were prepared from mesenteric lymph nodes (MLNs), lamina propria (LP), epithelium, spleen (SPL), lung and liver as previously described (24, 27, 28). Briefly, MLNs, spleen and liver were pressed and filtered through cell strainers. Small intestine (SI) and large intestine (LI) were harvested and Peyer's patches removed prior to process. LP and lung were digested with collagenase D and DNase I. LP, IEL, and liver lymphocytes were enriched by 40:75% Percoll density gradient centrifugation.

### Flow cytometry analysis

For surface staining, isolated cells were stained for flow cytometry with the following mAbs from Biolegend, eBioscience and/or TONBO: CD3 (145-2C11), CD4 (GK1.5 and RM4–5), CD8α (53-6.7), CD8? (YTS156.7.7), CD44 (IM7), CD45.1 (A20), CD45.2 (104), CD62L (MEL-14), CD69 (H1.2F3), CD90.1 (HIS51 or OX-7), and TCR H-Y (T3.70) in a conjugation with FITC, PE, PE-Cy5, PE-Cy7, APC, APC-Cy7 or PB. Propidium iodide (PI) (Sigma Aldrich) was used at 500 ng/ml of final concentration for staining of 1–5 × 10^6^ of cells to label dead cells. For intracellular staining, surface stained cells were fixed and permeabilized with BD cytofix/cytoperm according to manufacturer's protocol (BD Biosciences) and were stained with the following mAbs: IL-2 (JES6-5H4), IFN-γ (9D3.1C8), TNF-α (MP6-XT22) and Granzyme B (GB12). Flow cytometry samples were analyzed using a flow cytometer (LSR Fortessa and Canto-II; BD Biosciences) with DIVA software. Data were analyzed using Flowjo (Treestar).

### Immunohistochemistry

Small intestine was harvested and Peyer's patches were removed. Freshly collected tissues were “snap-frozen” in OCT (Leica) with liquid nitrogen. Tissue sections (6 μm in thickness) were prepared, air-dried, fixed for 10 min at 4°C in methanol (Merk). Cryosections were blocked for 30 min with biotin blocking solutions (Invitrogen), washed in PBS, and incubated overnight at 4°C with anti-Thy1.1 (OX-7) and anti-CD8α (53–6.7) antibodies (BioLegend). Sections were then washed with PBS and stained with DAPI. All slides were mounted with Prolong Antifade Reagent (Life technologies) and images were capture with Zeiss LSM 700 CLSM (confocal laser scanning microscope).

### Bacteria and virus infections

*Listeria monocytogenes* (LM) strain 10403s, carrying a recombinant internalin A (InIA) mutant, has been described in detail previously (29, 30). Briefly, B6 mice were infected with 5 × 10^10^ CFU *Listeria monocytes* (LM) InIA-OVA through oral gavage. For acute infections, B6 mice were infected i.p. with 2 × 10^5^ PFU of LCMV Armstrong (31).

### Administration of antibodies and/or cytokines *in vivo*

IL-2/anti-IL-2 complexes were prepared as previously described (12, 13). In brief, 1 μg of recombinant mouse IL-2 (PeproTech) was mixed with 5 μg of anti-IL-2 (S4B6) (BD Biosciences) and injected i.p daily for three consecutive days after adoptive cell transfer in SPF RAG^−/−^. For the IL-2 blocking experiments, 100 μg of anti-IL-2 (JES6-1A12) (Bio X Cell) and anti-IL-2 (S4B6) (BD Biosciences) was injected i.p. every other day for 7 days after adoptive cell transfer in SPF RAG^−/−^ and B6 hosts.

### Intravascular T cell staining

Following the previously published protocol (32), 3 μg of anti-Thy1.1 (OX-7) in 300 μl of PBS were injected i.v. into a mouse for intravascular staining of donor OT-I (Thy1.1) CD8^+^ T cells. The mice were killed 10 min after injection and collected the tissues. For discrimination of vascular and tissue donor cells, the cells were stained with *ex vivo* Abs including anti-Thy1.1 (HIS51).

### Statistical analysis

Results represent the mean ± SEM unless indicated otherwise. Statistical significance was determined by the unpaired Student's t test. Statistical analyses were performed using Prism GraphPad software v5.0. ^*^p < 0.05; ^**^p < 0.01; ^***^p < 0.001, ^****^p < 0.0001; ns, not significant).

## Results

### Spontaneous proliferation of polyclonal naïve T cells in RAG^−/−^ hosts

Given the well-known previous observations that polyclonal naïve CD4^+^ or CD8^+^ T cells undergo intense form of proliferative responses in a Rag-deficient host (15), which is referred to as spontaneous proliferation (SP), we sought to address whether and how this SP response of T cells influences their functional behavior and homeostasis during their reconstitution from lymphopenia. We thus first confirmed the prior notion that the SP occurs largely in an antigen-dependent manner with strong and fast rate of cell division kinetics.

**Figure 1:**
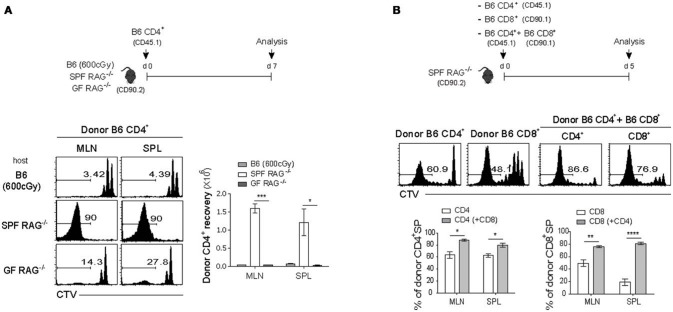
Polyclonal naïve CD4^+^ and CD8^+^ T cells undergo spontaneous proliferation in RAG^−/−^ hosts**. (A)** CTV-labeled naïve (Foxp3^−^ CD44^lo^ CD62L^hi^) (CD45.1) CD4^+^ T cells purified from Foxp3-eGFP mice were intravenously (i.v.) injected into irradiated (600cGy) B6, SPF RAG^−/−^ and GF RAG^−/−^ (CD90.2) hosts (1 × 10^6^ cells per mouse; top). Mesenteric lymph nodes (MLN) and spleen (SPL) of the recipient mice were analyzed on day 7 by flow cytometry for CTV dilution (bottom left) and donor cell recovery (bottom right). **(B)** CTV-labeled naïve (CD44^lo^ CD62L^hi^) CD4^+^ (CD45.1) and CD8^+^ (CD90.1) T cells were i.v injected either separately or together into SPF RAG^−/−^ (CD90.2) hosts (1 × 10^6^ cells for each subset per mouse; top). MLN and SPL of the recipient mice were analyzed on day 5 by flow cytometry for CTV dilution (middle) and percentage of spontaneous proliferation (SP) (bottom; mean ± SEM; *n* = 2–4 mice per group). Data are representative of at least three independent experiments. ^*^p < 0.05; ^**^p < 0.01; ^***^p < 0.001; ^****^p < 0.0001.

For this, FACS-purified CTV-labeled polyclonal naïve CD4^+^ T cells were adoptively transferred into three different lymphopenic hosts, namely C57BL/6 (B6) mice receiving sub-lethal doses (600 cGy) of irradiation and Rag1-deficient (RAG^−/−^) mice raised under the specific pathogen-free (SPF) or germ-free (GF) condition (Figure 1A, top). Donor cell division and recovery from the spleen (SPL) and mesenteric lymph nodes (MLN) were analyzed on day 7 after adoptive transfer by flow cytometry. As shown in Figure 1A, donor CD4^+^ T cells, as expected, exhibited only ~2–3 rounds of slow rate of cell division (i.e., un-gated CTV^+^ cells), referred to as lymphopenia-induced homeostatic proliferation (LIP) that is known to be dependent on TCR interaction with self-ligands and cytokine IL-7 (3, 7). In sharp contrast, cells transferred into SPF RAG^−/−^ hosts showed robust proliferative responses, as evidenced by the full dilution of CTV dye (i.e., gated CTV^−^ cells); however, these responses were abrogated substantially in GF RAG^−/−^ hosts, confirming the previous findings showing stringent dependence of the SP responses of polyclonal naïve CD4^+^ T cells on antigens derived from commensal microbiota (15). Unlike SP, the slower rate of LIP responses of donor cells was uninterrupted in the GF RAG^−/−^ hosts, level of which was similar to that of irradiated B6 hosts (Figure 1A, left; compare un-gated CTV^+^ cells in the top and bottom histogram). Thus, the recovery of donor cells was ~10-20-fold lower for the LIP responses in GF RAG^−/−^ and irradiated B6 hosts than those for the SP responses observed in SPF RAG^−/−^ hosts (Figure 1A, right). As for the SP of CD4^+^ T cells, polyclonal naïve CD8^+^ T cells from B6 mice also showed robust levels of SP, albeit at lower extent than CD4^+^ T cell SP, in SPF RAG^−/−^ hosts, but not in irradiated B6 hosts (Figure S1), which was also antigen-dependent because the SP response of CD8^+^ T cells was abolished in GF RAG^−/−^ hosts (data not shown).

Although the above data clearly confirmed the previously well-defined SP responses of either polyclonal naïve CD4^+^ or CD8^+^ T cells in SPF RAG^−/−^ hosts, interesting findings came from the experiments in which both naïve CD4^+^ and CD8^+^ T cells were co-transferred into these hosts. As shown in Figure 1B, in comparison to the proportion of SP responses after single transfer of either CD4^+^ or CD8^+^ T cells (63% ± 3.85 and 48% ± 2.95, respectively for MLN), we observed significantly enhanced SP responses of CD4^+^ T cells and to a greater extent CD8^+^ T cells on day 7 after co-transfer into SPF RAG^−/−^ hosts (80% ± 1.92 and 78% ± 1.20, respectively for MLN). These data suggest that there is a positive cross-talk between two distinct T cell compartments by which the SP response of one compartment is facilitated by those of another compartment during reconstitution period after adoptive co-transfer into SPF RAG^−/−^ hosts.

### Influence of the SP driven by polyclonal CD4^+^ T cells on the LIP of TCR Tg CD8^+^ T cells

Based on the above findings of such potential cross-talk between polyclonal naïve CD4^+^ and CD8^+^ T cells in SPF RAG^−/−^ hosts, we then addressed the question of whether and how the SP responses of two distinct lineages of naïve T cell populations may affect each of their homeostasis in this lymphopenic condition. We thus next sought to investigate the potential impact of the SP response driven by polyclonal naïve CD4^+^ T cell compartment on the typical slow rate of lymphopenia-induced homeostatic proliferation (LIP)—a response that is antigen-independent but IL-7-dependent—of naïve CD8^+^ T cells co-transferred into SPF RAG^−/−^ hosts.

**Figure 2:**
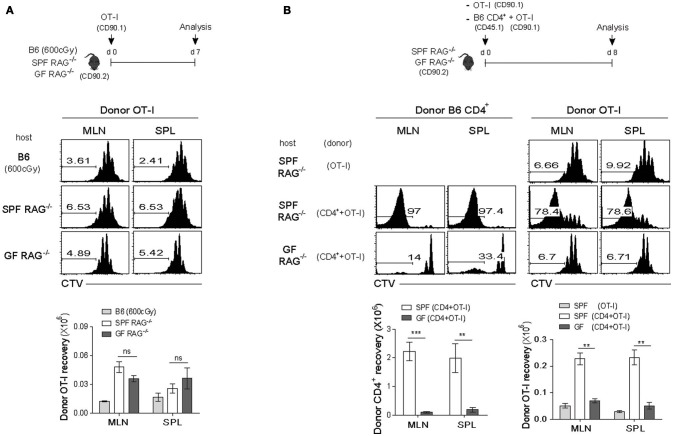
Rapid LIP response of monoclonal CD8^+^ T cells is associated with the strong SP response of polyclonal CD4^+^ T cells in RAG^−/−^ hosts. **(A)** CTV-labeled OT-I CD8^+^ T cells from OT-I.RAG^−/−^ mice (CD90.1) were injected into irradiated (600cGy) B6, SPF RAG^−/−^ and GF RAG^−/−^ (CD90.2) hosts (5 × 10^5^ cells per mouse; top) and then analyzed on day 7 by flow cytometry for CTV dilution (middle) and donor cell recovery (bottom). **(B)** CTV-labeled OT-I CD8^+^ T cells (CD90.1; 5 × 10^5^ cells) were i.v. injected either alone or along with polyclonal naïve CD4^+^ T cells (CD45.1; 1 × 10^6^ cells) into SPF RAG^−/−^ and GF RAG^−/−^ hosts (CD90.2; top). MLN and SPL of the recipient mice were then analyzed on days 7-8 by flow cytometry for CTV dilution (middle) and donor cell recovery (bottom). Data shown are the mean ± SEM (*n* = 5 mice per group) and are representative of four independent experiments. ^**^p < 0.01; ^***^p < 0.001; ns, not significant.

For this, we utilized monoclonal TCR transgenic (Tg) CD8^+^ T cells in order to avoid antigen-dependent SP response, which was observed for polyclonal B6 CD8^+^ T cells (Figure 1B), and validate the true influence of CD4^+^ T cell-driven strong SP response on the weak LIP response of monoclonal CD8^+^ T cells. Thus, naïve OT-I TCR Tg cells that are specific for H-2K^b^-restricted ovalbumin (OVA) 257-264 peptide were labeled with CTV and adoptively transferred into irradiated B6 (600 cGy), SPF RAG^−/−^, or GF RAG^−/−^ hosts and 7 days later, proliferative responses of donor OT-I cells were analyzed by flow cytometry (Figure 2A, top). As shown in Figure 2A, OT-I cells, unlike polyclonal CD8^+^ T cells, did not show robust SP responses, but instead did show only ~3–4 rounds of the typical slow-rate of LIP responses in SPF RAG^−/−^ hosts, similar to those seen in irradiated B6 or GF RAG^−/−^ hosts. Donor cell recoveries in the spleen were also similar in all three lymphopenic recipients, although there was a modest difference in the mLN (Figure 2A, bottom). These data thus confirmed the prior notion that the SP response observed in RAG^−/−^ hosts is dependent on commensal microbial antigens and thus detectable only for polyclonal, but not monoclonal, CD8^+^ T cells.

Based on the above results showing prevalent occurrence of the LIP but not the SP upon adoptive transfer with OT-I cells into SPF RAG^−/−^ hosts, we then investigated the influence of the SP response driven by polyclonal CD4^+^ T cells on the LIP response of OT-I cells. Thus, a mixture of FACS-purified, CTV-labeled naïve B6 CD4^+^ T cells and OT-I CD8^+^ T cells was transferred into SPF RAG^−/−^ or GF RAG^−/−^ hosts, and as a control, OT-I cells alone were also transferred into SPF RAG^−/−^ hosts, and then analyzed on day 8 for their proliferation and cell recovery (Figure 2B, top). Here, the surprising finding was that, in marked contrast to the typical slow rate of LIP after single transfer of OT-I cells alone, these cells showed much faster and greater levels of proliferative responses when co-transferred with B6 CD4^+^ T cells into SPF RAG^−/−^ hosts (Figure 2B, middle). More importantly, such intense responses of OT-I cells seen in SPF RAG^−/−^ hosts were not observed in GF RAG^−/−^ hosts even in the presence of B6 CD4^+^ T cells being co-transferred, resulting in the poor donor cell recovery (Figure 2B, bottom). Careful analysis for the kinetics of these robust proliferative responses revealed that the appearance of the fast-dividing OT-I cells was apparent from day 4 after co-transfer with B6 CD4^+^ T cells into SPF but not GF RAG^−/−^ hosts, a time point of which CD4^+^ T cells also began to show significant levels of SP responses (Figure S2), suggesting a role of B6 CD4^+^ T cells undergoing SP.

Together, these findings strongly suggest that the SP response of polyclonal CD4^+^ T cells in SPF RAG^−/−^ hosts plays a role in promoting LIP response of co-transferred monoclonal (and also polyclonal) CD8^+^ T cells, leading to the alteration of the speed and degree of their proliferation in a chronic lymphopenic environment.

### Influence of the polyclonal CD8^+^ T cell-derived SP on LIP of TCR Tg CD8^+^ T cells

**Figure 3:**
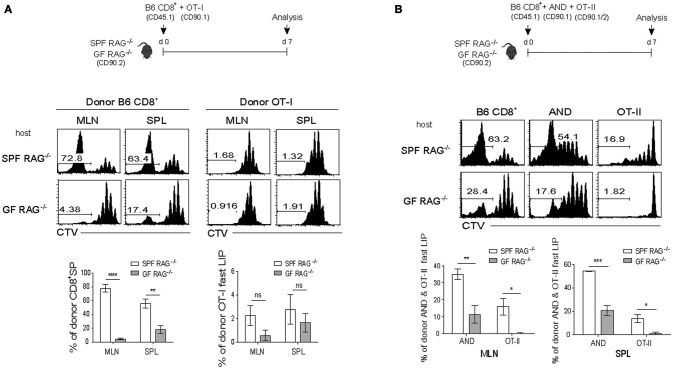
The SP response of polyclonal CD8^+^ T cells promotes the rapid LIP response of CD4^+^ but not CD8^+^ T cells. **(A)** A mixture of CTV-labeled polyclonal naïve CD8^+^ T cells (CD45.1; 1 × 10^6^ cells) and OT-I CD8^+^ T cells (CD90.1; 5 × 10^5^ cells) was injected into SPF RAG^−/−^ and GF RAG^−/−^ hosts (CD90.2; top). MLN and SPL of the mice were then analyzed on day 7 by flow cytometry for CTV dilution (middle) and percentages of the SP of polyclonal CD8^+^ T cells (CD8^+^ SP; bottom left) and of the fast-dividing lymphopenia-induced proliferation (LIP) of OT-I cells (OT-I fast LIP; bottom right). **(B)** A mixture of CTV-labeled polyclonal naïve CD8^+^ T cells (CD45.1; 2 × 10^6^ cells) and either AND CD4^+^ T cells from AND.RAG^−/−^ mice (CD90.1; 1 × 10^6^ cells) or OT-II CD4^+^ T cells from OT-II.RAG^−/−^ mice (CD90.1/90.2; 1 × 10^6^ cells) was injected into SPF RAG^−/−^ and GF RAG^−/−^ hosts (CD90.2; top) and then analyzed on day 7 by flow cytometry for CTV dilution (middle) and percentages of the fast-dividing LIP of AND and OT-II CD4^+^ T cells (bottom). Data shown are the mean ± SEM (*n* = 3–4 mice per group) and are representative of three independent experiments. ^*^p < 0.05; ^**^p < 0.01; ^***^p < 0.001; ^****^p < 0.0001; ns, not significant.

Given the above stimulatory effect of the SP response of polyclonal CD4^+^ T cells on the LIP of monoclonal OT-I cells in SPF RAG^−/−^ hosts, we next addressed whether the similar enhancing effect is also observed with the SP response of polyclonal CD8^+^ T cells, because these cells also showed strong antigen-dependent SP in SPF RAG^−/−^ hosts (Figure 1B and Figure S1). For this, a mixture of FACS-purified, CTV-labeled naïve B6 CD8^+^ T cells and OT-I cells was transferred into SPF RAG^−/−^ or GF RAG^−/−^ hosts (Figure 3A, top). At day 7 after adoptive transfer, as expected, B6 CD8^+^ T cells showed robust SP responses in SPF but not GF RAG^−/−^ hosts (Figure 3A, middle and bottom left). Surprisingly, however, such SP responses of B6 CD8^+^ T cells completely failed to induce strong LIP responses of the co-transferred OT-I cells in SPF RAG^−/−^ hosts, as evidenced by the lack of CTV full-diluted, fast-dividing cells in these recipients, which was comparable to those seen in GF RAG^−/−^ hosts (Figure 3A, middle and bottom right).

The rather unexpected results from these co-transfer experiments led us to speculate a possible involvement of clonal competition within the same lineage of donor CD8^+^ T cell populations presumably for self-ligands, and suggest that SP response of polyclonal T cells in SPF RAG^−/−^ hosts may result in a diverse degree of LIP differing from one clone to another.

### Impact of TCR affinity for self-ligands on the strong LIP response mediated by the SP

The above clonal competition for self-antigens within the same lineage of CD8^+^ T cell pools is of particular importance to determine the rate and magnitude of their LIP in lymphopenic hosts, as has been demonstrated by previous reports (6, 25, 33). It was thus intriguing for us to test whether the SP response of polyclonal B6 CD8^+^ T cells has an enhancing effect on the LIP of monoclonal CD4^+^, if not CD8^+^, TCR Tg cells—in which clonal competition for self-pMHC ligands is avoided—similar to the effect observed with B6 CD4^+^ T cells and OT-I CD8^+^ T cells in SPF RAG^−/−^ hosts (Figure 2B).

For this, FACS-purified, CTV-labeled naïve B6 CD8^+^ T cells were co-transferred with a mixture of AND TCR Tg (specific for I-E^k^-restricted pigeon cytochrome C 81-104 peptide) and OT-II TCR Tg (specific for I-A^b^-restricted Ova 323-339 peptide) naïve CD4^+^ T cells into SPF RAG^−/−^ or GF RAG^−/−^ hosts and 7 days later, the mice were analyzed for donor cell proliferations by flow cytometry (Figure 3B, top). In marked contrast to the OT-I CD8^+^ cells (Figure 3A), AND and to a lesser extent OT-II CD4^+^ cells showed a significant increase in the proportion of their fast-rate of LIP by B6 CD8^+^ T cells in SPF but not GF RAG^−/−^ hosts (Figure 3B). Here, the greater effect on the LIP of AND cells than that of OT-II cells likely reflects the difference in their relative TCR affinity for self-ligands; thus, the LIP response was apparently faster and greater for T cells with a high affinity TCR (i.e., AND cells) than those with a low affinity TCR (i.e., OT-II cells) (34).

As for the above AND and OT-II CD4^+^ cells, similar difference was observed with two distinct CD8^+^ TCR Tg cells, namely OT-I vs. HY (specific for H-2D^b^-restricted male antigen-derived peptide), in which their intrinsic TCR affinity for self-ligands is much lower in HY cells than in OT-I cells (5, 6, 35). Thus, the results again clearly showed that the enhancing effect on the LIP was much greater for OT-I cells than for HY cells, when these cells were co-transferred with B6 CD4^+^ T cells into SPF RAG^−/−^ hosts (Figure S3A). Likewise, the enhancing effect on the LIP of OT-I cells was also further confirmed together with co-transfer of P14 TCR Tg CD8^+^ T cells (specific for H-2D^b^-restricted lymphocytic choriomeningitis virus glycoprotein 33-41 peptide) into SPF RAG^−/−^ hosts (Figure S3B). These results thus suggest that the effect we observed is not OT-I-specific but broadly applicable for different monoclonal CD8^+^ T cell populations with variable degrees depending on their relative TCR affinity for self-ligands.

Together, these findings indicate that the SP response of either polyclonal CD4^+^ or CD8^+^ T cells in SPF RAG^−/−^ hosts contributes to promoting the faster and greater LIP response of CD8^+^ or CD4^+^ T cells, respectively, in a TCR-self-MHC dependent manner.

### Effect of Ag-specific CD4^+^ T cell activation on the CD8^+^ T cell LIP in acute lymphopenic conditions

The above data so far pointed out the unique ability of polyclonal T cells to promote the strong LIP of TCR Tg cells only when the former induces robust SP response in a chronic severe lymphopenic host such as SPF RAG^−/−^ mice. Because the SP response in these hosts was commensal microbial antigen-dependent and thus failed to occur in a GF condition, it is possible that the above phenomenon is limited to a particular condition of SPF RAG^−/−^ mice rather than a general occasion of typical lympho-depleted mice through irradiation, cytotoxic drugs, or T cell-depleting antibodies.

**Figure 4:**
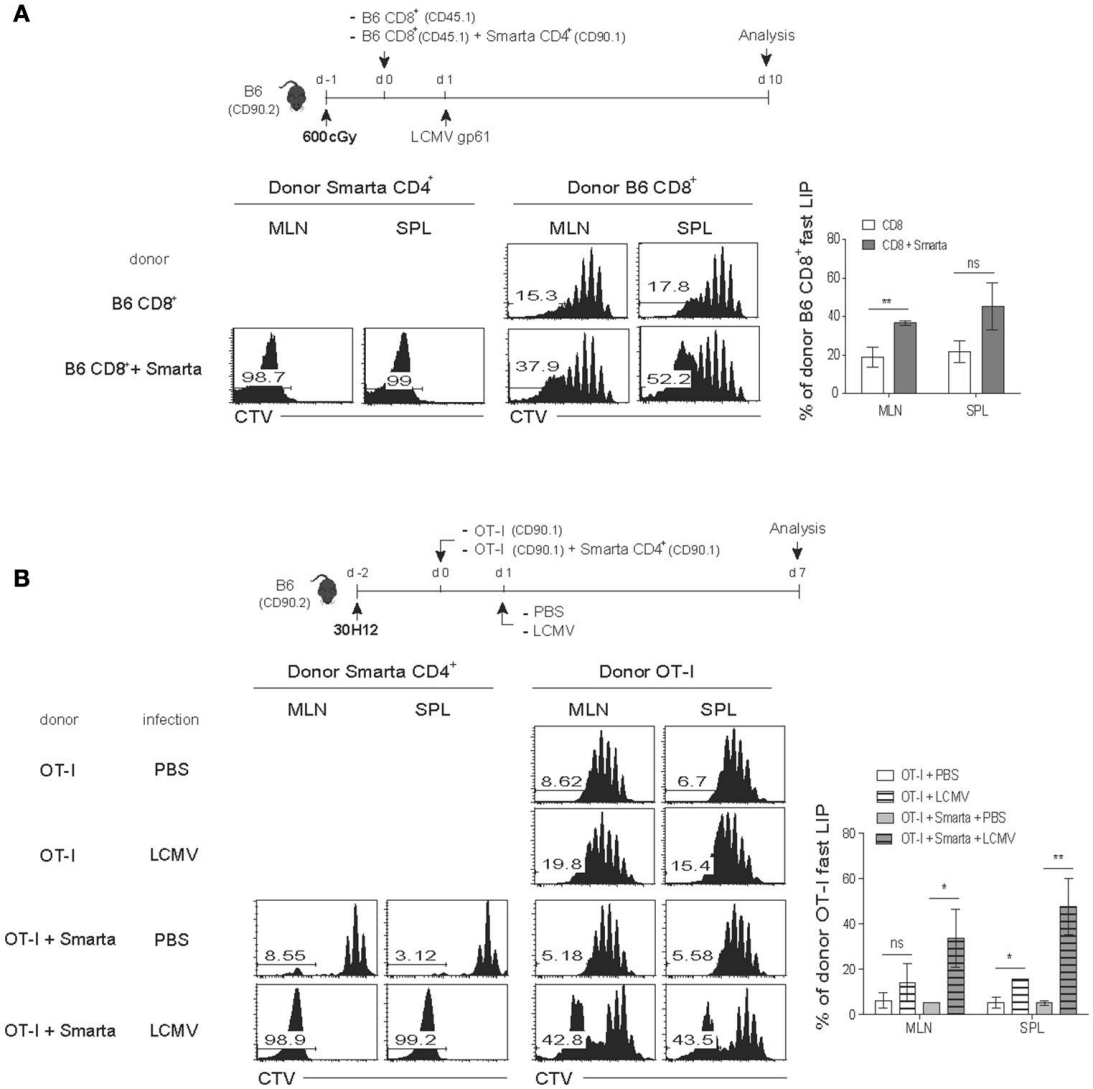
Antigen-induced SP response of CD4^+^ T cells enhances the fast-dividing LIP of CD8^+^ T cells in various lymphopenic settings. **(A)** B6 mice (CD90.2) were treated with a sub-lethal dose of irradiation (600 cGy) 1 day before cell transfer and then injected i.v. with CTV-labeled polyclonal naïve CD8^+^ T cells (CD45.1; 5 × 10^5^ cells) either alone or along with naïve SMARTA CD4^+^ T cells from SMARTA TCR Tg mice (CD90.1; 5 × 10^4^ cells) and 1 day later, immunized intraperitoneally (i.p.) with LCMV peptide GP_61−80_ (top). MLN and SPL were analyzed on day 7 by flow cytometry for CTV dilution (bottom left two panels) and percentages of the fast LIP of donor CD8^+^ T cells (bottom right). **(B)** B6 mice (CD90.2) were injected i.p. with anti-Thy1.2 mAb (30H12) 2 days before cell transfer and then injected i.v. with CTV-labeled OT-I CD8^+^ T cells (CD90.1; 5 × 10^5^ cells) either alone or along with SMARTA CD4^+^ T cells (CD90.1; 5 × 10^4^ cells) and 1 day later, injected i.p. either with PBS or with LCMV Armstrong (2 × 10^5^ PFU; top). MLN and SPL were analyzed on day 7 by flow cytometry for CTV dilution (bottom left two panels) and percentages of the fast LIP of donor OT-I cells (bottom right). Data shown are the mean ± SEM (*n* = 3-4 mice per group) and are representative of at least three independent experiments. ^*^p < 0.05; ^**^p < 0.01; ns, not significant.

We thus sought to address this issue of whether antigen-induced strong T cell responses would also facilitate the rate and intensity of LIP of naïve CD8^+^ T cells (either from B6 and TCR Tg mice). For this, we generated two different lymphopenic settings derived from normal B6 mice by treatment of either a sub-lethal dose of irradiation (Figure 4A) or a monoclonal antibody against Thy1.2 for depleting host T cell compartment (anti-Thy1.2 mAb; clone 30H12; Figure 4B). In the first lymphopenic setting, normal B6 hosts receiving irradiation (600 cGy) were adoptively transferred with B6 CD8^+^ T cells either alone or together with SMARTA TCR Tg CD4^+^ T cells (specific for I-A^b^-restricted lymphocytic choriomeningitis virus glycoprotein 61-80 peptide; LCMV GP61) and 1 day later, were immunized with LCMV GP61 peptide antigen (Figure 4A, top); noted that the peptide antigen must be used here as a stimulus for SMARTA cells, because LCMV infection was lethal for the irradiated mice. Thus, while donor B6 CD8^+^ T cells showed only a typical slow rate of LIP response when transferred alone with peptide injection, interesting finding was that these CD8^+^ cells showed significantly elevated levels of rapidly dividing cells when SMARTA cells were co-transferred and activated with its cognate peptide antigen LCMV GP61 (Figure 4A, bottom). Here, the rather smaller increase of the fast-dividing donor B6 CD8^+^ T cells in irradiated B6 hosts with activated SMARTA cells (~37–52%; Figure 4A) relative to those of donor OT-I cells in SPF RAG^−/−^ hosts with B6 CD4^+^ T cells (~78–80%; Figure 2B) might reflect a diverse range of heterogeneity in polyclonal CD8^+^ T cell pools for their TCR affinity to self-ligands. In light of this view, we indeed found that the extent of the fast-rate of LIP driven by peptide-stimulated SMARTA cells in irradiated B6 hosts was much higher for CD5^hi^ donor B6 CD8^+^ T cells than for CD5^lo^ counterparts co-transferred (~76–79% vs. ~13–14%, respectively; Figure S4). The data thus suggest a role of the activated CD4^+^ T cells (here SMARTA cells) in promoting the rate and degree of antigen-independent LIP response of naïve CD8^+^ T cells even in an irradiation-induced, acute lymphopenic condition.

To further confirm the above findings, we then utilized the second alternative approach in which normal B6 mice were injected with anti-Thy1.2 mAb (30H12) to acutely deplete host T cell compartment. The mAb-treated mice were then adoptively transferred with OT-I CD8^+^ T cells either alone or along with SMARTA CD4^+^ T cells, followed by either being un-infected or infected with LCMV to stimulate the latter SMARTA cells specifically (Figure 4B, top). Consistent with the results from the above irradiation-induced lymphopenic settings, the increased proportion of rapidly dividing OT-I cells was prominent with LCMV infection and subsequent activation of co-transferred SMARTA cells, whereas there was no such increase of OT-I cell proliferation by SMARTA cells without LCMV infection (~43% vs. ~5–6%, respectively; Figure 4B, bottom two rows). As a control, there was only a slow rate of LIP of OT-I cells without SMARTA cells regardless of LCMV infection (Figure 4B, middle two rows); however, noted that, albeit at a smaller portion, LCMV infection appears to have a tendency of slight increase of OT-I cell LIP in this T-depleted condition, presumably due to some innate responses derived from residual host-derived, non-T cell populations.

The above effects on the OT-I cell LIP driven by the antigen-stimulated SMARTA cells were all observed in acute lymphopenic conditions (Figures 4A,B). Importantly, however, these effects were not detected in normal lympho-replete hosts; thus, upon LCMV infection without 30H12 mAb-induced lymphopenia, both the slow- and the fast-responding LIP of OT-I cells were severely decreased (Figure S5), therefore highlighting a stringent requirement of lymphopenia. Collectively, these findings strongly suggest that the above phenomenon seen in SPF RAG^−/−^ hosts is not due to their unique environment of chronic lymphopenia. Instead, the elevated levels of the fast-rate LIP of naïve CD8^+^ T cells can efficiently occur under various forms of acute lymphopenic conditions if two key requirements are provided, namely CD4^+^ T cells and their specific cognate antigens capable of stimulating these cells.

### Role of IL-2 in promoting strong LIP response in acute lymphopenic hosts

The above findings that the stimulated CD4^+^ T cells under acute lymphopenic settings led to the faster and greater LIP of CD8^+^ T cells prompted us to elucidate its underlying mechanisms and raise the question of how this is regulated and which factor is involved. Based on the fact that the LIP of naïve CD8^+^ T cells is known to be antigen-independent and largely driven by cytokines, especially IL-7 (or both IL-7 and to a lesser extent IL-15 for naïve CD4^+^ T cells) (7, 10), it is possible that the enhanced LIP response was just a mere reflection of greatly increased levels of these cytokines, presumably accompanied by the antigen-dependent activation of CD4^+^ T cells in this condition.

**Figure 5:**
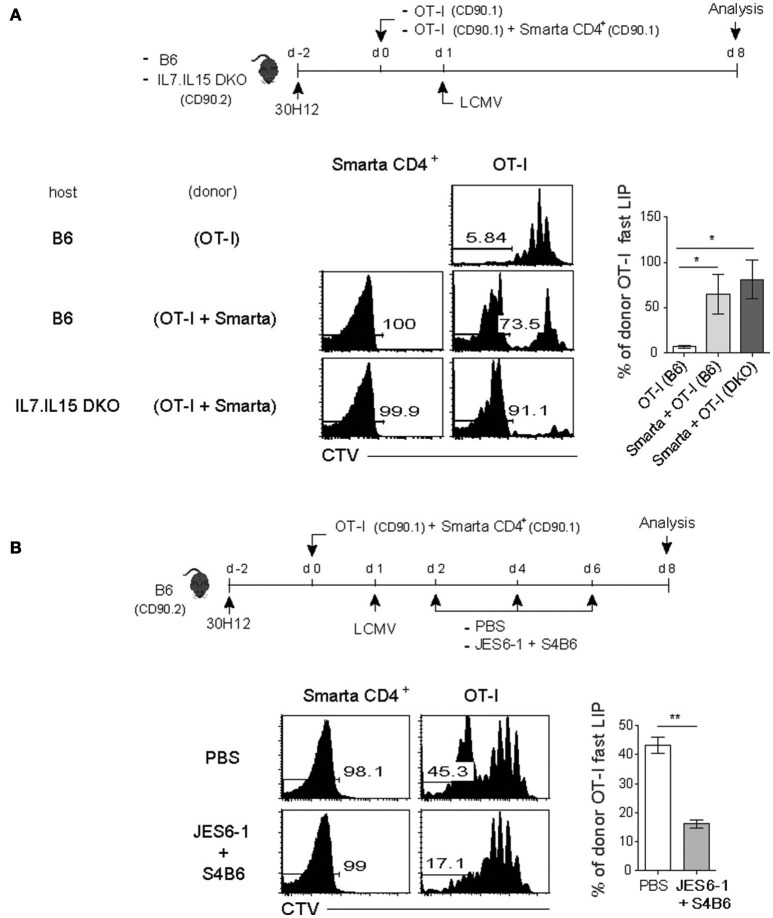
The fast-dividing LIP response is independent of both IL-7 and IL-15 but is dependent on IL-2**. (A)** Wild-type (B6) and IL-7.IL-15 double knock-out (DKO) mice (CD90.2) were injected i.p. with anti-Thy1.2 mAb (30H12) 2 days before cell transfer (top). These mice were then injected i.v. with CTV-labeled OT-I CD8^+^ T cells (CD90.1; 5 × 10^5^ cells) either alone or along with SMARTA CD4^+^ T cells (CD90.1; 5 × 10^4^ cells) and 1 day later, infected i.p. with LCMV Armstrong (2 × 10^5^ PFU). CTV dilution (bottom left) and percentages of the fast LIP of donor OT-I cells (bottom right) were analyzed on day 8 by flow cytometry. **(B)** B6 mice treated with 30H12 mAb were injected i.v. with a mixture of CTV-labeled OT-I CD8^+^ (CD90.1; 5 × 10^5^ cells) and SMARTA CD4^+^ T cells (CD90.1; 5 × 10^4^ cells) and then infected i.p. with LCMV Armstrong (2 × 10^5^ PFU; top). These mice were injected i.p. either with PBS or anti-IL-2 mAbs (two clones; JES6-1 and S4B6) at the indicated time points (top). CTV dilution (bottom left) and percentages of the fast LIP of donor OT-I cells (bottom right) were analyzed on day 8 by flow cytometry. Data shown are the mean ± SEM (*n* = 3 mice per group) and are representative of at least three independent experiments. ^*^p < 0.05; ^**^p < 0.01.

We therefore tested this possibility of a role of the elevated amounts of IL-7 and/or IL-15 as a mechanism of promoting the antigen-independent strong LIP of CD8^+^ T cells after LCMV infection. For this, a mixture of SMARTA CD4^+^ and OT-I CD8^+^ T cells was adoptively transferred into the aforementioned T-depleted lymphopenic B6 mice (using 30H12 mAb treatment) of either wild-type (WT) or double knock-out (DKO) for both IL-7 and IL-15, and 1 day later, the mice were infected with LCMV and analyzed on day 7 by flow cytometry (Figure 5A, top). Here, the notable finding was that the enhancing effect on the LIP of OT-I cells was prominent with LCMV-activated SMARTA cells for both WT and DKO hosts compared to that of OT-I cells without SMARTA cells (Figure 5A, bottom), implying a role of different factor(s) other than IL-7 and IL-15.

In an attempt for searching the key factor(s), we then tested a possible role of IL-2 because this cytokine is mainly produced from T cells after antigenic stimulation. Moreover, especially for naïve CD8^+^ T cells, IL-2 is known to induce an intense form of antigen-independent, rapid proliferative response in both lympho-deplete and even lympho-replete conditions (13). To address a role of IL-2, the 30H12-treated B6 mice were adoptively transferred with a mixture of SMARTA and OT-I cells and 1 day later, infected with LCMV in the presence or absence of anti-IL-2 mAbs (JES6-1 and S4B6 clones) for blocking *in vivo* IL-2, and then analyzed on day 8 by flow cytometry (Figure 5B, top). Here, the result was surprising; while the OT-I cells co-transferred with SMARTA cells showed the elevated proportion of the fast-dividing LIP, these cells failed to do so after LCMV infection along with IL-2 blockade (Figure 5B, bottom). The data thus suggest that the enhancing effect of the antigen-activated CD4^+^ T cells on the LIP of CD8^+^ T cells was associated at least in part with *in vivo* activity of IL-2.

### Role of IL-2 in enhancing strong LIP response in SPF RAG^−/−^ hosts

The implication of the above results with IL-2 blockade is that the IL-2 may act as a key factor and is produced by LCMV-activated SMARTA CD4^+^ T cells, a level of which is perhaps sufficient to promote the rapid and robust form of antigen-independent LIP of co-transferred OT-I CD8^+^ T cells in T-depleted hosts. Given the close similarity in the requirement of antigen-induced CD4^+^ T cell activation, we reasoned that the enhancing effect on the LIP of OT-I cells observed in SPF RAG^−/−^ hosts (Figure 2B) might also be mediated via a mechanism that is dependent on IL-2 presumably produced from activated CD4^+^ T cells undergoing SP in these hosts.

**Figure 6:**
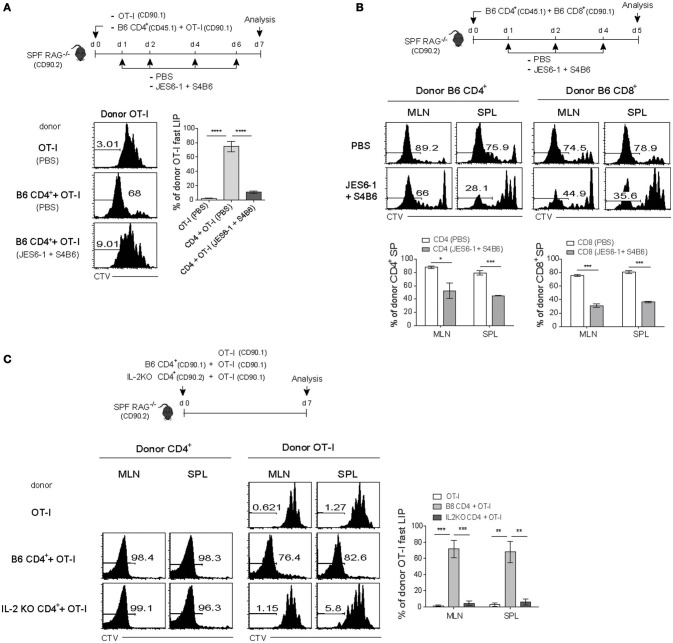
The fast-dividing LIP is mediated by IL-2 produced as a result of the SP response of CD4^+^ T cells. **(A)** CTV-labeled OT-I CD8^+^ T cells (CD90.1; 5 × 10^5^ cells) either alone or along with polyclonal naïve CD4^+^ T cells (CD45.1; 1 × 10^6^ cells) were injected i.v. into SPF RAG^−/−^ hosts (CD90.2; top). The mice were then injected i.p. either with PBS or with anti-IL-2 mAbs (JES6-1 and S4B6) at the indicated time points (top). CTV dilution (bottom left) and percentages of the fast LIP of donor OT-I cells (bottom right) were analyzed on day 7 by flow cytometry. **(B)** A mixture of CTV-labeled polyclonal naïve CD4^+^ (CD45.1; 1 × 10^6^ cells) and CD8^+^ T cells (CD90.1; 1 × 10^6^ cells) was injected i.v. into SPF RAG^−/−^ hosts (CD90.2) and then treated i.p. with either PBS or anti-IL-2 mAbs (JES6-1 and S4B6) at the indicated time points (top). MLN and SPL were analyzed on day 5 by flow cytometry for CTV dilution (middle) and percentages of the fast LIP of donor CD4^+^ and CD8^+^ T cells (bottom). **(C)** CTV-labeled OT-I CD8^+^ T cells (CD90.1; 5 × 10^5^ cells) either alone or along with polyclonal naïve CD4^+^ T cells (1 × 10^6^ cells) purified from either wild-type B6 (CD90.1) or IL-2-deficient (IL-2 KO; CD90.2) mice were injected i.v. into SPF RAG^−/−^ hosts (CD90.2; top). MLN and SPL of the recipient mice were then analyzed on day 7 by flow cytometry for CTV dilution (bottom left two panels) and percentages of the fast LIP of donor OT-I cells (bottom right). Data shown are the mean ± SEM (*n* = 3 mice per group) and are representative of three independent experiments. ^*^p < 0.05; ^**^p < 0.01; ^***^p < 0.001, ^****^p < 0.0001.

We therefore tested again this possibility and the results were indeed the case with the following two observations: (1) a decrease and (2) an increase in the fast-dividing LIP by blocking or enhancing *in vivo* IL-2 activity, respectively (Figure 6A and Figure S6A). For this, SPF RAG^−/−^ mice were adoptively transferred with either OT-I cells alone or a mixture of OT-I cells and B6 CD4^+^ T cells, and then untreated or treated either with anti-IL-2 mAbs for blocking IL-2 (Figure 6A, top) or with IL-2 as a form of IL-2 and anti-IL-2 (S4B6) immune complex known to enhance IL-2 activity *in vivo* (Figure S6A) (12, 36). Here, the result was that OT-I cells co-transferred with B6 CD4^+^ T cells failed to show the fast-dividing LIP after IL-2 blockade, while undergoing the slow-rate of LIP similar to those seen in OT-I cells transferred alone (Figure 6A, bottom). Conversely, however, the lack of the fast-rate LIP of OT-I cells transferred alone was completely restored after treatment with IL-2/anti-IL-2 complex, to the level comparable to those seen in OT-I cells co-transferred with B6 CD4^+^ T cells (Figure S6A). Moreover, these IL-2/anti-IL-2 complexes also induced the faster and greater LIP of OT-I cells even when co-transferred with competing B6 CD8^+^ T cells into SPF RAG^−/−^ hosts (Figure 3A and Figure S6B). Besides these findings with OT-I cells, we also obtained similar data for polyclonal B6 CD8^+^ and CD4^+^ T cells co-transferred into SPF RAG^−/−^ hosts with or without blocking IL-2 (Figure 6B); here, again, the *in vivo* IL-2 blockade led to ~26-62% of significant reduction of the fast-dividing CD4^+^ and CD8^+^ T cells in these hosts.

We further confirmed the data from the above blocking experiments with anti-IL-2 mAb to validate the role of IL-2 produced by CD4^+^ T cells undergoing SP in SPF RAG^−/−^ hosts. For this, we first generated bone marrow (BM) chimera reconstituted with a 50:50 ratio of WT and IL-2^−/−^ BM cells to avoid spontaneous T cell activation due to autoimmunity in IL-2^−/−^ mice (37) and 8 weeks later, naïve CD4^+^ T cells derived from WT and IL-2^−/−^ BM were isolated from the chimeric mice (Figure S6C). OT-I cells were then adoptively transferred alone or co-transferred with either the WT or the IL-2^−/−^ CD4^+^ T cells into SPF RAG^−/−^ hosts and analyzed on day 7 by flow cytometry (Figure 6C, top). While the fast-dividing LIP of OT-I cells was apparent with WT CD4^+^ T cells, this response was totally abolished with IL-2^−/−^ CD4^+^ T cells despite their SP response was relatively intact (Figure 6C, bottom). Consistent with this finding, our data from additional transfer experiments also revealed that rapidly dividing activated CD4^+^ T cells in SPF RAG^−/−^ hosts indeed show a profound synthesis of intracellular IL-2 (and IFN-γ) after short-term *in vitro* restimulation, a level of which was much higher than that of OT-I cells co-transferred (Figure S7).

Together, all these findings strongly support the notion that IL-2 is produced from polyclonal CD4^+^ (and CD8^+^) T cells that are activated as a result of antigen-dependent SP response in SPF RAG^−/−^ hosts, which then acts as a key player for promoting the rapid and robust form of antigen-independent, but IL-2-dependent LIP of monoclonal CD8^+^ T cells in these hosts.

### Effector activity of IL-2-driven strong LIP response against bacterial infection

Given the above data showing the strong proliferation of CD8^+^ T cells driven by IL-2 that is produced from antigen-stimulated CD4^+^ T cells co-transferred into either a chronic or an acute lymphopenic host, we next sought to address whether these robust form of antigen-independent, IL-2-driven response is functionally relevant for immune responses.

**Figure 7:**
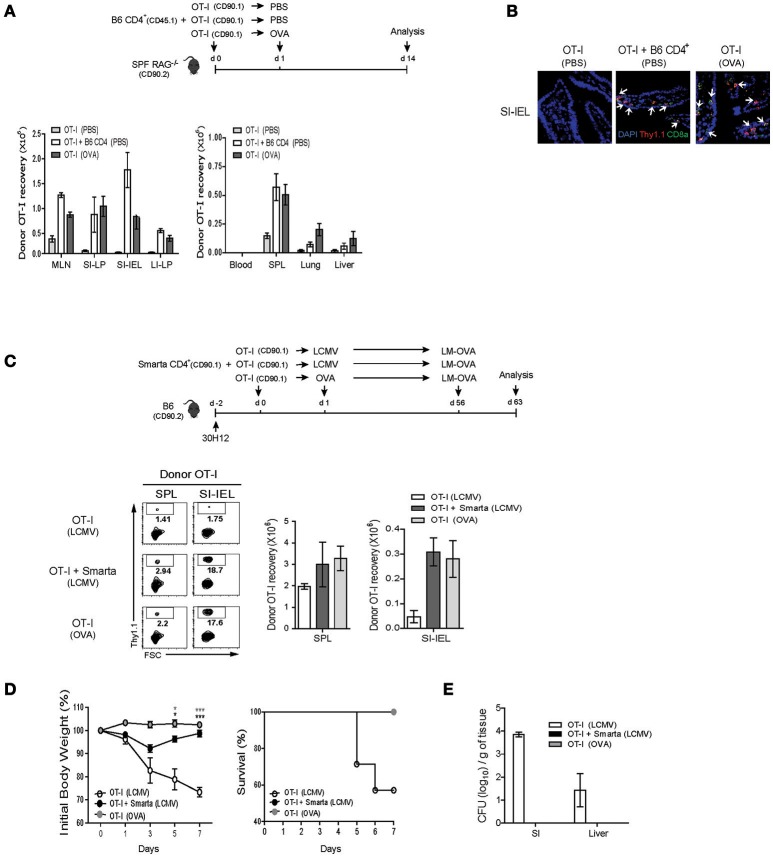
IL-2-driven strong LIP response leads to the protective immunity against bacterial infection. **(A,B)** OT-I CD8^+^ T cells (CD90.1; 5 × 10^5^ cells) were injected i.v. either alone or along with polyclonal naïve CD4^+^ T cells (CD90.1 or CD45.1; 1 × 10^6^ cells) into SPF RAG^−/−^ hosts (CD90.2; top). The mice were then either left with PBS injection or immunized i.p. with OVA protein (100 μg/mouse) and analyzed on day 14 (top). The various lymphoid organs indicated were analyzed for donor cell recovery by flow cytometry (**A**; bottom) and for donor cell migration to the small intestine (SI) by immunofluorescence histochemistry with antibodies specific for DAPI (blue), anti-Thy1.1 (red), and anti-CD8α (green; **B)**. Data shown in **(A)** are the mean ± SEM (*n* = 4–6 mice per group). Representative immunofluorescence images from three independent experiments are shown **(B)**. **(C–E)** B6 mice (CD90.2) were treated i.p. with anti-Thy1.2 mAb (30H12) 2 days before cell transfer and then injected i.v. with OT-I CD8^+^ T cells (CD90.1; 5 × 10^5^ cells) either alone or along with SMARTA CD4^+^ T cells (CD90.1; 5 × 10^4^ cells) and l day later, immunized i.p. either with LCMV Armstrong (2 × 10^5^ PFU) or with OVA protein (100 μg/mouse; top). At 56 days after adoptive transfer, the mice were then challenged with OVA-expressing *Listeria monocytogenes* (LM-OVA) via oral gavage (5 × 10^10^ CFU; top). The mice were analyzed on day 7 post-challenge for donor OT-I recovery from the SPL and SI (for intraepithelial lymphocytes; IEL) by flow cytometry **(C)**, and were monitored at the indicated time points for body weight loss and survival (D), and also measured for bacterial counts in the SI and liver by plaque assay **(E)**. Data shown are the mean ± SEM (*n* = 5 mice per group) and are representative of three independent experiments. ^*^p < 0.05; ^***^p < 0.001.

For this, SPF RAG^−/−^ hosts were adoptively transferred with either a mixture of OT-I cells and B6 CD4^+^ T cells or as a control OT-I cells alone, and for the latter control group, were either un-immunized or immunized with OVA antigen, and then analyzed on day 7 by flow cytometry (Figure 7A, top). Here, as expected, donor OT-I cells co-transferred with B6 CD4^+^ T cells showed robust expansion and thus their recoveries from blood, SPL, lung and liver were significantly greater than those of OT-I cells transferred alone without immunization (Figure 7A, bottom left). Importantly, the degree of expansion and recovery of OT-I cells were as near similar when co-transferred with B6 CD4^+^ T cells as those of OT-I cells when transferred alone with OVA stimulation, and in particular, were also prominent in the gut-associated lymphoid tissues such as mLN, small, and large intestine (SI and LI, respectively) (Figure 7A, bottom right). Such preferential recovery of donor OT-I cells from the SI, especially intraepithelium (IEL) was also further confirmed by immunofluorescent tissue staining (Figure 7B). Furthermore, in line with the greater expansion, we found that the OT-I cells co-transferred with B6 CD4^+^ T cells into SPF RAG^−/−^ hosts exhibited a high expression of granzyme B as well as an ability to produce IFN-γ and TFN-α upon short-term *in vitro* restimulation, characteristics of differentiation into functional effector cells (Figure S8A).

Based on the appearance of functional effector CD8^+^ cells driven by IL-2 after co-transfer with B6 CD4^+^ T cells into SPF RAG^−/−^ hosts, we then investigated whether the similar events of effector differentiation would occur and also develop into functional memory cells in the aforementioned acute T cell-depleted hosts with LCMV infection, a phenomenon that is also likely to be IL-2 dependent (Figure 5B). For this, 30H12-treated, T cell-depleted B6 mice were adoptively transferred with OT-I cells either alone or along with SMARTA cells, and then immunized with either LCMV or as a control OVA antigen (only for hosts receiving OT-I cells alone) (Figure 7C, top). At 2 months later, the mice were infected via oral gavage with internalin A (InlA)-mutated *Listeria monocytogenes* expressing OVA antigen (LM-OVA; Figure 7C, top), a mutant strain that is restricted to the intestinal infection through gut epithelial cells (29, 30). Here, at day 7 after LM-OVA challenge, the percentage and recovery of donor OT-I cells that had been primed with LCMV-stimulated SMARTA cells were as prominent in the SPL and to a much greater extent SI-IEL as those of OT-I cells that had been primed by OVA antigen, but much greater than those of OT-I cells that had been primed irrelevantly with LCMV in the absence of SMARTA cells (Figure 7C, bottom). Likewise, the OT-I cells from the former two groups of recipients also showed much higher ability to synthesize granzyme B and produce IFN-γ and TFN-α after *in vitro* restimulation (Figure S8B). Most importantly, in line with such prominent memory recall responses, OT-I cells that had been primed with LCMV-activated SMARTA cells resulted in the strong protective responses against lethal doses of LM-OVA challenges, comparable to those of OT-I cells that had been primed with OVA antigen, as evidenced by the smaller body weight reduction, higher survival rates, and lesser bacterial counts than those of control OT-I cells that had been un-primed (Figures 7D,E).

Collectively, these data indicate that the IL-2-driven, robust LIP response of CD8^+^ T cells that is associated with antigen-dependent activation of CD4^+^ T cells under lymphopenic conditions is accompanied by efficient generation of and differentiation into effector and memory cells that are functional for protecting hosts from pathogenic infections.

## Discussion

Unlike the slow rate of lymphopenia-induced homeostatic proliferation (LIP), the rapid and robust form of proliferative responses has been documented for naïve T cells particularly in a chronic lymphopenic host such as RAG^−/−^ and TCRβ^−/−^ (and also CD3ε^−/−^) mice (15, 17). Although this response (called as spontaneous proliferation; SP) is known to be antigen-dependent—an antigen derived from commensal microbiota, precise nature of this response and its impact on homeostasis and function for the responding T cells during their recovery from lymphopenia remain incompletely understood. In the present study, we addressed these issues and demonstrated that, upon adoptive transfer of polyclonal B6 naïve CD4^+^ and CD8^+^ T cells into SPF but not GF RAG^−/−^ hosts, strong SP response of these cells affects the intensity and the tempo of the responding T cells, especially for those of undergoing antigen-independent LIP. Thus, the resulting LIP response in SPF RAG^−/−^ hosts was rapid and intense, and was influenced by TCR affinity for self-ligands and most importantly, heavily dependent on IL-2 that is produced from activated T cells undergoing antigen-dependent SP. Notably, these observations were not limited to a unique environment of chronic lymphopenia but rather broadly applicable for various other acute lymphopenic conditions with two crucial requirements, namely antigen-dependent T cell activation and availability of relatively high amounts of IL-2. As a consequence, T cells undergoing IL-2-driven strong LIP showed a full capacity to differentiate into functional effector and memory cells that can provide a protective response against pathogenic bacterial infection.

The exact nature of stimuli for driving the strong SP response in RAG^−/−^ hosts is unclear but a number of evidence revealed that this response mainly depends on a strong TCR signal via its engagement with a cognate antigen—presumably from commensal microbial components—and also costimulatory signal through CD28 (15, 18, 38, 39). Such dependency explains why the SP response occurs only with polyclonal but not monoclonal T cell populations, and is undetectable in GF RAG^−/−^ hosts or severely reduced even in SPF RAG^−/−^ hosts after treatment with antibiotics (15, 18). This notion therefore seems to fit well with the idea that the SP response by polyclonal naïve CD4^+^ or CD8^+^ T cells in SPF RAG^−/−^ hosts is a reflection of strong responsiveness of a few, albeit rare, clones that have specificity to a variety of commensal-derived peptide antigens. In fact, it has been shown that for CD4^+^ T cells, the SP response is impaired in chronic lymphopenic mice lacking MHC-II expression (15, 17) but unimpaired in mice either being treated with mAb for blocking IL-7 or lacking IL-7 expression (7, 40, 41). Given this notion, our data showing enhanced SP response of B6 CD4^+^ and CD8^+^ T cells co-transferred into SPF RAG^−/−^ hosts compared to those of either CD4^+^ or CD8^+^ T cells transferred separately were rather unexpected (Figure 1B), because these cells were unlikely to share TCR specificity for cognate antigens and they were subjected to strong activation independently in an antigen-specific manner.

Based on the findings from the above co-transfer experiments, it was tempting to speculate that the SP response is perhaps influenced at least in part by a factor other than TCR engagement with cognate antigens. This prediction was indeed true and supported by the following two surprising observations: the rapid and robust “SP-like” proliferative responses of (1) OT-I TCR Tg CD8^+^ T cells co-transferred with B6 CD4^+^ T cells and (2) AND TCR Tg CD4^+^ T cells co-transferred with B6 CD8^+^ T cells into SPF RAG^−/−^ hosts (Figures 2B, 3B). Because there are no cognate antigens specific for OT-I and AND cells, such robust proliferations of these cells we observed in SPF RAG^−/−^ hosts appear to rule out a role of antigen-dependent signals through OT-I or AND TCR *per se*. However, despite the lesser importance of antigen-specific TCR engagement, the robust responses of TCR Tg cells in SPF RAG^−/−^ hosts still depend on a covert TCR signal derived from its contacts with self-ligands. Thus, the reduced proliferative responses were apparent with monoclonal or polyclonal T cells of lower TCR affinity for self-ligands, e.g., HY or OT-II cells and B6 CD5^lo^ cells transferred into SPF RAG^−/−^ hosts (compared to those of OT-I or AND cells and B6 CD5^hi^ cells, respectively; Figure 3B and Figures S3A, 4). In this respect, the observed self-dependence of these TCR Tg cells and relative difference in their proliferative response in SPF RAG^−/−^ hosts closely resemble those seen with these cells in acute lymphopenic hosts (7, 8), although the intensity and tempo of the proliferative responses are distinctly different. Hence, the logical explanation for the proliferative response of monoclonal TCR Tg cells driven by polyclonal T cells in SPF RAG^−/−^ hosts is that this phenomenon falls into the same category as the typical IL-7-driven LIP response in that the response occurs (1) only in lymphopenic conditions; depends (2) on TCR engagement with self-ligands; and is (3) independent of cognate antigenic stimulation. But the difference is that the former is much faster and stronger than the latter LIP and is driven by a combination of two important stimuli, namely a tonic TCR self-reactivity and presumably a much more potent cytokine than IL-7.

In an attempt to search for the latter cytokine factor that has a stimulatory activity, IL-2 is the most suitable candidate. This prediction is based on our findings that the robust LIP of TCR Tg cells in SPF RAG^−/−^ hosts is stringently dependent on the presence of polyclonal B6 T cells and their strong activation, likely inducing effector cells capable of producing IL-2. In fact, we and others have previously shown that IL-2 acts as a potent stimulator for naïve CD8^+^ T cells by itself even in the absence of antigenic stimulation both *in vitro* and *in vivo* (35, 42). Moreover, this cytokine—either alone or along with IL-15—was shown to induce much intense form of proliferative responses for naïve T cells in lympho-deplete or even lympho-replete hosts (12, 35, 43), the intensity and tempo of which are similar to those of the strong LIP response we observed in SPF RAG^−/−^ hosts. Because these previous studies, however, utilized an experimental setting in which naïve T cells are exposed to a supraphysiological level of IL-2 in a rather unphysiological condition, a role of IL-2 was needed to be validated in our system. In this regard, two critical questions arise: (1) does IL-2 indeed act as a key stimulatory factor? and (2) are polyclonal B6 T cells that are activated and proliferated in SPF RAG^−/−^ hosts a major source for *in vivo* IL-2 production? These were indeed the case and clearly supported by a series of our *in vivo* data showing a decrease of the robust LIP response of OT-I cells by IL-2 blockade (Figure 6A), an increased response by IL-2 administration (Figures S6A,B), and finally an impaired response when co-transferred with IL-2^−/−^ CD4^+^ T cells (Figure 6C). Although a stimulatory role of IL-2 was better highlighted with TCR Tg cells (here OT-I cells), it should be noted, however, that the effect by IL-2 was also prominent for polyclonal B6 T cells (Figure 6B). Thus, the reduction of the strong proliferative responses by IL-2 blockade was apparent for both B6 CD8^+^ and to a lesser extent CD4^+^ T cells. Why the B6 CD4^+^ T cells were less effective for the IL-2 blockade is unclear. Whether this is a reflection of less contribution of IL-2-driven LIP yet more reliance on antigen-driven SP for CD4^+^ T cells than for CD8^+^ T cells remains to be addressed.

Based on the above effect of IL-2, it seems conceivable that the typical SP response of polyclonal B6 T cells in SPF RAG^−/−^ hosts largely consists of at least two different forms of proliferation, namely an antigen-dependent “true” SP as well as an antigen-independent and IL-2-dependent “bystander” LIP, with its strength and rate of proliferation akin to those of the SP. In light of this notion, it should be taken into caution that the SP response reported in some previous studies utilizing co-transfer experiments of polyclonal CD4^+^ and CD8^+^ T cells into the same chronic lymphopenic hosts might include such a bystander component of IL-2-driven rapid LIP. Because this response in these hosts occurs in a manner independent of TCR engagement with cognate antigens, T cells (especially for CD8^+^ T cells) would be strongly responding and proliferating even in a situation where MHC-I expression is limited or absent if MHC-II expression is intact for CD4^+^ T cells to drive their SP and IL-2 production. In fact, a previous study has shown that, when transferred with an unseparated mixture of naïve CD4^+^ and CD8^+^ T cells, the strong proliferative response of the latter population is found even in MHC-I-deficient TCRβ^−/−^ hosts and concluded that this phenomenon is MHC-II-, but not MHC-I-, dependent (17). In this situation, however, determining whether the observed response of CD8^+^ T cells in an MHC-I-lacking environment would reflect the effect of IL-2 produced from the co-transferred antigen-activated CD4^+^ T cells will be interesting.

How IL-2 can induce such a robust SP-like bystander response is unclear. Previously we have addressed this issue and demonstrated that IL-2 (or IL-15) has a unique ability to drive activation and proliferation of naïve T cells, particularly CD8^+^ T cells, via a mechanism dependent on the density of lipid rafts on the T cell membrane (35). In this study, a relatively high concentration of IL-2 *in vitro* could induce a rapid membrane relocalization and clustering of IL-2Rβ chain (CD122) into the membrane micro-domains of lipid rafts, leading to the enhanced activation and amplification of IL-2R and its downstream signal transduction pathways, including activation of JAK/STAT, ERK, and PI3K/AKT signaling pathways (35). Hence, the implication from these studies is that naïve CD8^+^ T cells (and to a lesser extent CD4^+^ T cells) are able to undergo rapid SP-like responses only when these cells are exposed to IL-2 at high concentrations and that this response may occur in any situation where IL-2 is increased at sufficiently high levels *in vivo*. Inducing IL-2 production may be easily achieved by strong antigenic stimulation of naïve T cells, yet reaching an effective concentration at levels sufficient for inducing robust SP-like response seems less easy largely because of shorter half-life of *in vivo* IL-2 and its rapid consumption by overwhelming numbers of T cells being expanded during antigen-specific immune responses. Indeed, we found that, upon adoptive transfer of OT-I cells into normal lympho-replete B6 hosts, potent antigenic stimulation of either polyclonal B6 (host-derived) or monoclonal SMARTA CD4^+^ T cells (co-transferred) triggered by LCMV infection fails to induce IL-2-driven strong proliferative responses of OT-I cells (Figure S5). The result, however, was totally different when such antigenic stimulation occurs in a lympho-deplete condition, resulting in the robust IL-2-dependent SP-like response of OT-I cells (Figures 4, 5). Our findings therefore provide strong support for the notion that such robust proliferative responses of naïve T cells can be easily driven by relatively increased levels of *in vivo* IL-2 produced from antigen-stimulated T cells in both situations of chronic and acute lymphopenia.

Our study presented here does not rule out the possibility that the IL-2 may function indirectly through CD4^+^ T cells or DCs rather than direct action on CD8^+^ T cells (19). Here, the possible scenario is that IL-2 may modulate DCs to induce co-stimulatory molecules such as B7.1 (CD80) and B7.2 (CD86) that can enhance a stimulatory activity of DCs or, alternatively, would help to promote CD4^+^ T cell activation and subsequent upregulation of co-stimulatory molecule, such as CD40L, engagement of which then results in maturation and stimulation of otherwise immature DCs. As a consequence of such IL-2 conditioning, the resulting DCs would then facilitate the robust proliferative responses of bystander CD8^+^ T cells. In fact, despite some controversial results (44), the upregulation of CD80 and CD86 on DCs has been documented in RAG^−/−^ mice (19); here, the expression of these molecules on DCs was higher in RAG^−/−^ mice than in normal B6 mice, and was largely attributed to the lack of CD4^+^ T regulatory (Treg) cells, which are known to restrain upregulation of co-stimulatory molecules and maturation/activation of steady-state DCs via CTLA4 (45–47). More importantly, DC costimulation was even further upregulated in RAG^−/−^ mice when adoptively transferred with naïve CD4^+^ T cells. Although IL-2 has not been considered as a modulatory factor for DCs in this study, the enhanced DC expression of co-stimulatory molecules observed with CD4^+^ T cell transfer into RAG^−/−^ hosts did not seem to require the activity of IL-2—presumably produced from CD4^+^ T cells transferred—on DCs with mainly two reasons: First, the enhancement of CD80 and CD86 expression on DCs in RAG^−/−^ mice was not detected with *in vivo* administration of IL-2 delivered as a form of IL-2 and anti-IL-2 immune complexes (19), for which we also confirmed in our study (data not shown). Second, similar to the results observed with CD4^+^ T cell transfer into RAG^−/−^ hosts, the enhanced expression of DC costimulatory molecules has also been reported in mice lacking IL-2 or its receptors (CD25 and CD122), which is known to be accompanied by spontaneous activation of conventional CD4^+^ CD25^−^ T cells resulting from the lack of Treg suppression in these mice (48). Hence, although we favor the view that IL-2 is dispensable for inducing the enhanced DC expression of co-stimulatory molecules, the additional experiments—e.g., using adoptive transfer with CD25- or CD122-deficient OT-I cells into RAG^−/−^ hosts—will be necessary to provide strong evidence for a direct stimulatory role of IL-2 on CD8^+^ T cells.

Whether these observations with increased levels of IL-2 have any particular relevance in a normal physiological condition remains to be addressed. In this respect, it has been shown that, similar to responses in RAG^−/−^ hosts, naïve CD4^+^ T cells also undergo the rapid and intense form of SP response upon adoptive transfer into neonatal hosts (neonates at 1–3 days of age) (49). Therefore, it will be of interesting to investigate whether some, if not all, of the SP response of naïve T cells (especially for CD8^+^ T cells) that occur during a neonatal period is perhaps IL-2-dependent and how these cells would behave for their homeostasis and function throughout an adult life. With regard to the functional aspect of IL-2-driven T cell responses, we showed that these cells after the intense proliferative responses (both in RAG^−/−^ hosts and in lympho-depleted B6 hosts with LCMV infections) are able to fully differentiate into effector and memory cells that are functional for controlling pathogenic infections at levels equivalent to those of antigen-induced effector/memory cells (Figure 7). Therefore, it seems clear that these findings, together with additional future studies, would provide better understanding of the precise nature of the strong SP response of polyclonal naïve T cells in chronic lymphopenic hosts and of previously unappreciated role of IL-2 in regulating their homeostasis and functional responses.

In summary, besides a role of enhanced DC costimulation (19), a clear stimulatory role of IL-2 is apparent for naïve CD8^+^ T cells in SPF RAG^−/−^ hosts. We show here in this study that this IL-2-driven stimulatory effect occurs only in a particular situation where CD4^+^ T cells co-exist and their strong antigen-dependent activation is induced under various lymphopenic environments. Although the phenomenon observed in these particular conditions seems less physiological, our findings nevertheless would have an implication for the development of therapeutic interventions against cancer, especially for those of using pre-conditioning regime of lymph-depletion prior to adoptive T cell transfer-based immunotherapy (50–54).

## Ethics statement

This research was approved by the Institutional Animal Care and Use Committees (IACUC) of the Pohang University of Science and Technology (2013–01–0012). Mouse care and experimental procedures were performed in accordance with all institutional guidelines for the ethical use of non-human animals in research and protocols from IACUC of the Pohang University of Science and Technology.

## Author contributions

JK, JL, and KC performed experiments. JK, CS, and J-HC designed experiments and analyzed and interpreted the data. S-WH, KK, JS, and S-HI contributed to this study with valuable discussion and critical comments. JK and J-HC wrote the manuscript.

### Conflict of interest statement

The authors declare that the research was conducted in the absence of any commercial or financial relationships that could be construed as a potential conflict of interest.
