# Supplementary material for: Spontaneous Proliferation of CD4+ T Cells in RAG-Deficient Hosts Promotes Antigen-Independent but IL-2-Dependent Strong Proliferative Response of Naïve CD8+ T Cells
Source: Front Immunol. 2018 Aug 23;9:1907. doi: 10.3389/fimmu.2018.01907 (PMC6116856; doi:10.3389/fimmu.2018.01907)
Supplement: Supplementary file 1 [file Data_Sheet_1.docx]

Supplementary Material

**Spontaneous proliferation of CD4^+^ T cells in RAG-deficient hosts promotes antigen-independent but IL-2-dependent strong proliferative response of naïve CD8^+^ T cells**

Juhee Kim^1,2^, Jun Young Lee^1,2^, Kyungjin Cho^1,2^, Sung-Wook Hong^1,2^, Kwangsoon Kim^1,2^, Jonathan Sprent^3^, Sin-Hyeog Im^1,2^, Charles D. Surh^1,2^ and Jae-Ho Cho^1,2^.

^1^Academy of Immunology and Microbiology, Institute for Basic Science, Pohang 790-784, Korea.

^2^Department of Integrative Biosciences and Biotechnology, Pohang University of Science and Technology, Pohang 790-784, Korea.

^3^Immunology Division, Garvan Institute of Medical Research, Darlinghurst, New South Wales 2010, Australia.

**Correspondence:**

Charles D. Surh: csurh@ibs.re.kr

Jae-Ho Cho**:** [jhcho90@ibs.re.kr](mailto:jhcho90@ibs.re.kr)

**Supplementary Figures**

**Figure S1**

**
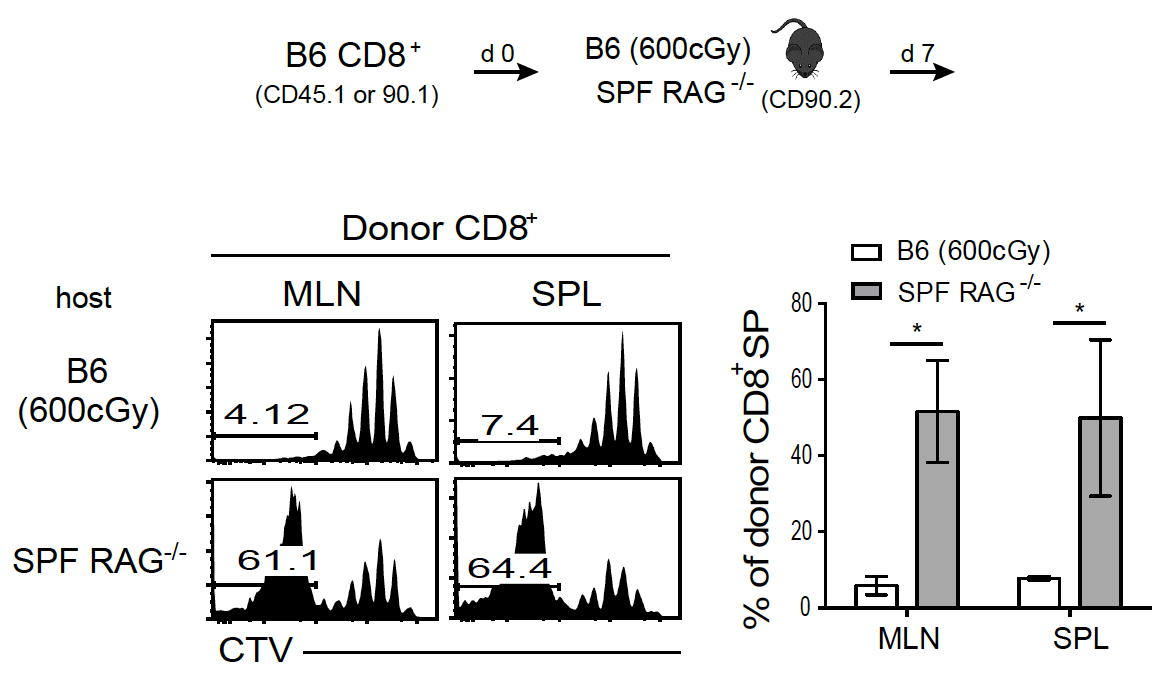
**

**Figure S1**. **Polyclonal naïve CD8^+^ T cells undergo spontaneous proliferation in RAG^−/−^ hosts.** CTV-labeled naïve (CD44^lo^ CD62L^hi^) CD8^+^ T cells purified from B6.PL (CD90.1) or B6.SJL (CD45.1) mice were intravenously (i.v.) injected into irradiated B6 (600 cGy) and SPF RAG^−/−^ hosts (CD90.2; 1 × 10^6^ cells per mouse; top). Mesenteric lymph nodes (MLN) and spleen (SPL) of the recipient mice were analyzed on day 7 by flow cytometry for CTV dilution (bottom left) and spontaneous proliferation (bottom right). Data shown are the mean ± SEM (*n* = 2-4 mice per group) and are representative of at least three independent experiments. **p* < 0.05.

**Figure S2**


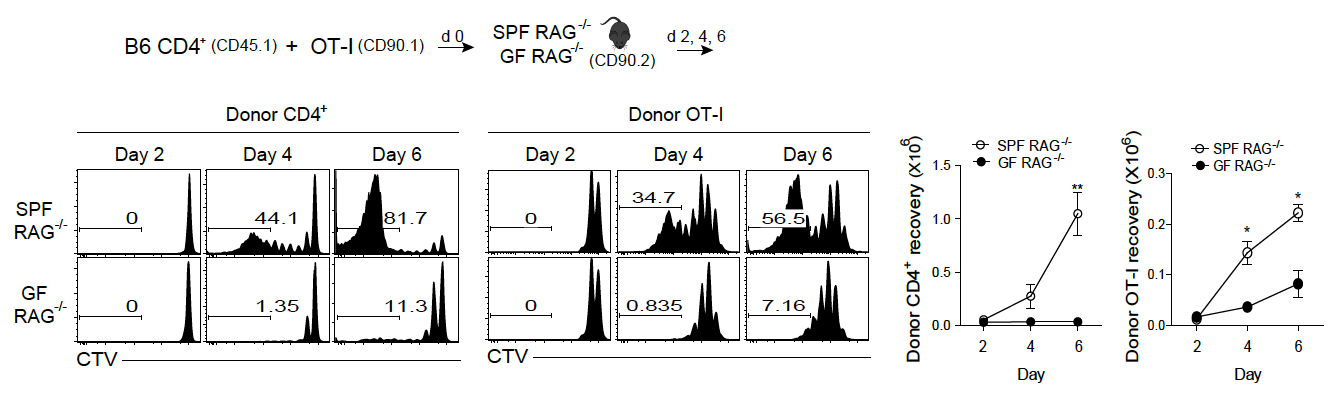


**Figure S2. Kinetics of proliferative responses of polyclonal CD4^+^ and monoclonal CD8^+^ T cells in RAG^−/−^ hosts.** A mixture of CTV-labeled polyclonal naïve CD4^+^ (CD45.1; 1 × 10^6^ cells) and OT-I CD8^+^ T cells (CD90.1; 5 × 10^5^ cells) was injected i.v. into SPF RAG^−/−^ and GF RAG^−/−^ hosts (CD90.2, top). Mice were then analyzed at the indicated time points by flow cytometry for CTV dilution (bottom left two panels) and total donor cell recovery (bottom right). Data shown are the mean ± SEM (*n* = 2-3 mice per group) and are representative of two independent experiments. **p* < 0.05; ***p* < 0.01.

**Figure S3**


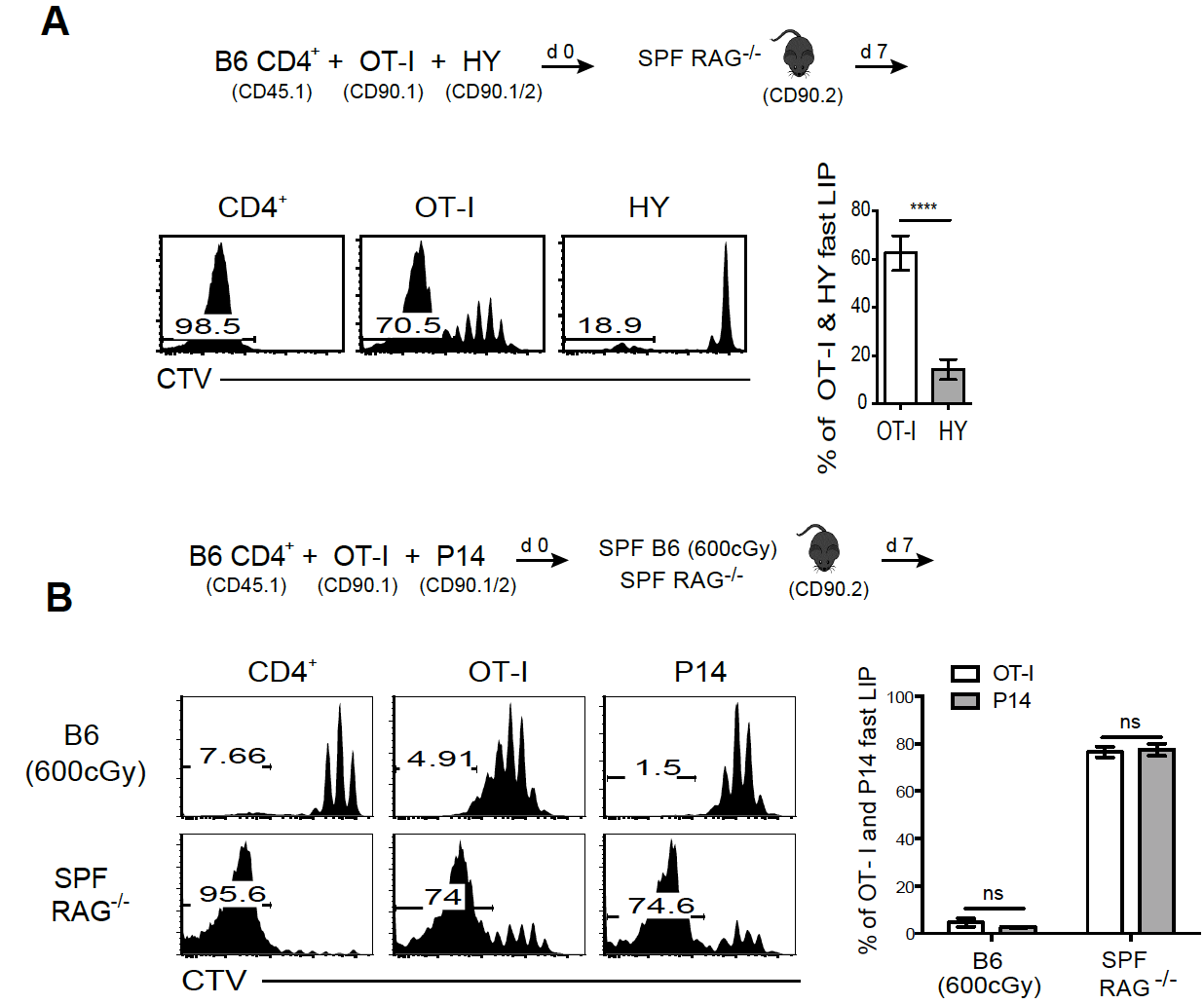


**Figure S3**. **The degree of the fast-dividing LIP is influenced by the relative TCR affinity for self-ligands. (A)** A mixture of CTV-labeled polyclonal naïve CD4^+^ T cells (CD45.1; 1 × 10^6^ cells) and OT-I CD8^+^ T cells from OT-I.RAG^−/−^ mice (CD90.1; 5 × 10^5^ cells) and HY CD8^+^ T cells from HY mice (CD90.1/90.2; 5 × 10^5^ cells) was injected into SPF RAG^−/−^ hosts (CD90.2; top) and then analyzed on day 7 by flow cytometry for CTV dilution (bottom left) and percentages of the fast-dividing LIP of OT-I and HY CD8^+^ T cells (bottom right). **(B)** A mixture of CTV-labeled polyclonal naïve CD4^+^ T cells (CD45.1; 1 × 10^6^ cells) and OT-I CD8^+^ T cells (CD90.1; 5 × 10^5^ cells) and P14 CD8^+^ T cells (CD90.1/90.2; 5 × 10^5^ cells) was injected into irradiated B6 (600 cGy) and SPF RAG^−/−^ hosts (CD90.2; top) and then analyzed on day 7 by flow cytometry for CTV dilution (bottom left) and percentages of the fast-dividing LIP of OT-I and P14 CD8^+^ T cells (bottom right). Data shown are the mean ± SEM (*n* = 3 mice per group) and are representative of three independent experiments. *****p* < 0.0001.

**Figure S4**

**
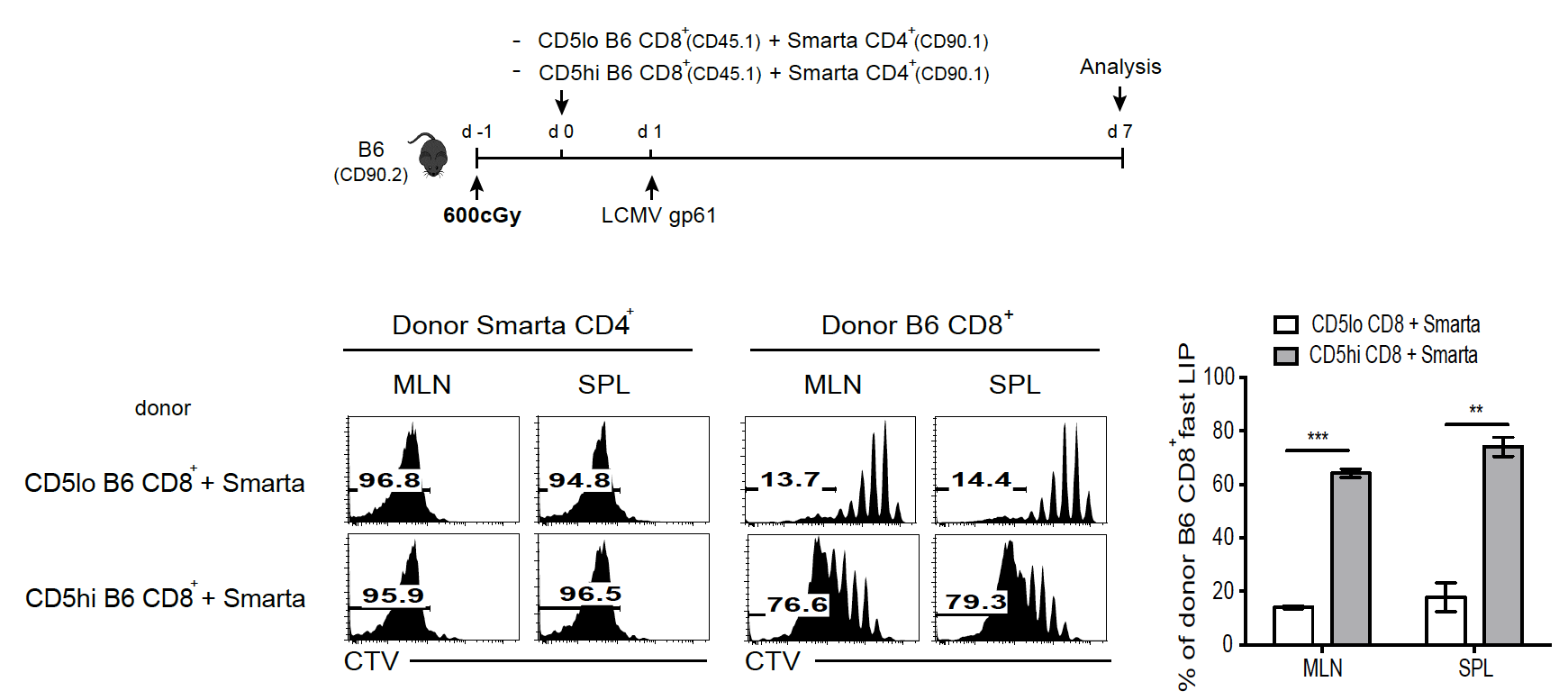
**

**Figure S4**. **The difference in the degree of the fast-dividing LIP between CD5^lo^ and CD5^hi^ B6 CD8^+^ T cells.** B6 mice (CD90.2) were treated with a sub-lethal dose of irradiation (600 cGy) 1 day before cell transfer and then injected i.v. with a mixture of CTV-labeled SMARTA CD4^+^ T cells (CD90.1; 5 × 10^4^ cells) and either polyclonal naïve CD5^lo^ or CD5^hi^ CD8^+^ T cells (CD45.1; 5 × 10^5^ cells) and 1 day later, immunized with LCMV peptide GP_61-80_ (top). MLN and SPL were analyzed on day 7 by flow cytometry for CTV dilution (bottom left two panels) and percentages of the fast-rate LIP of donor B6 CD8^+^ T cells (bottom right).

**Figure S5**


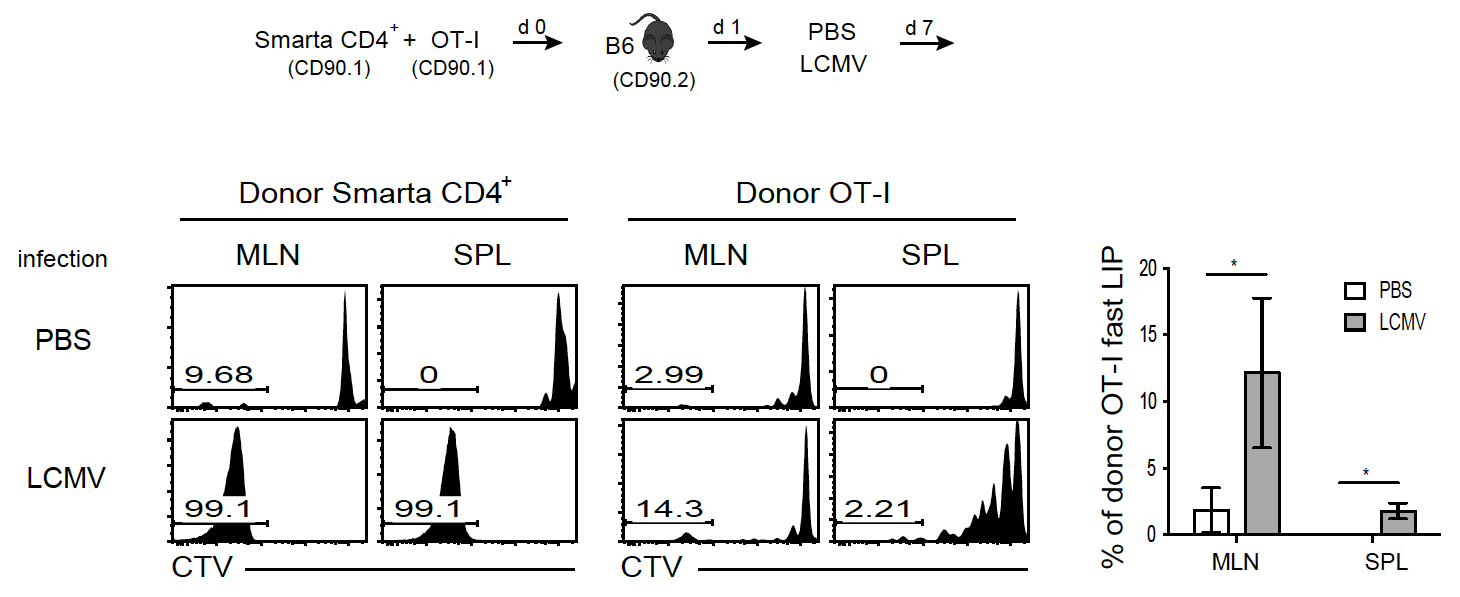


**Figure S5**. **Lymphopenia is a critical requirement for T cells to undergo the fast-dividing LIP.** B6 mice (CD90.2) were injected i.v. with CTV-labeled OT-I CD8^+^ T cells (CD90.1; 5 × 10^5^ cells) and SMARTA CD4^+^ T cells (CD90.1; 5 × 10^4^ cells) and 1 day later, injected i.p. either with PBS or with LCMV Armstrong (2 × 10^5^ PFU; top). MLN and SPL of the mice were analyzed on day 7 by flow cytometry for CTV dilution (bottom left two panels) and percentages of the fast LIP of donor OT-I cells (bottom right). Data shown are the mean ± SEM (*n* = 3-4 mice per group) and are representative of at least three independent experiments. **p* < 0.05.

**Figure S6**

**
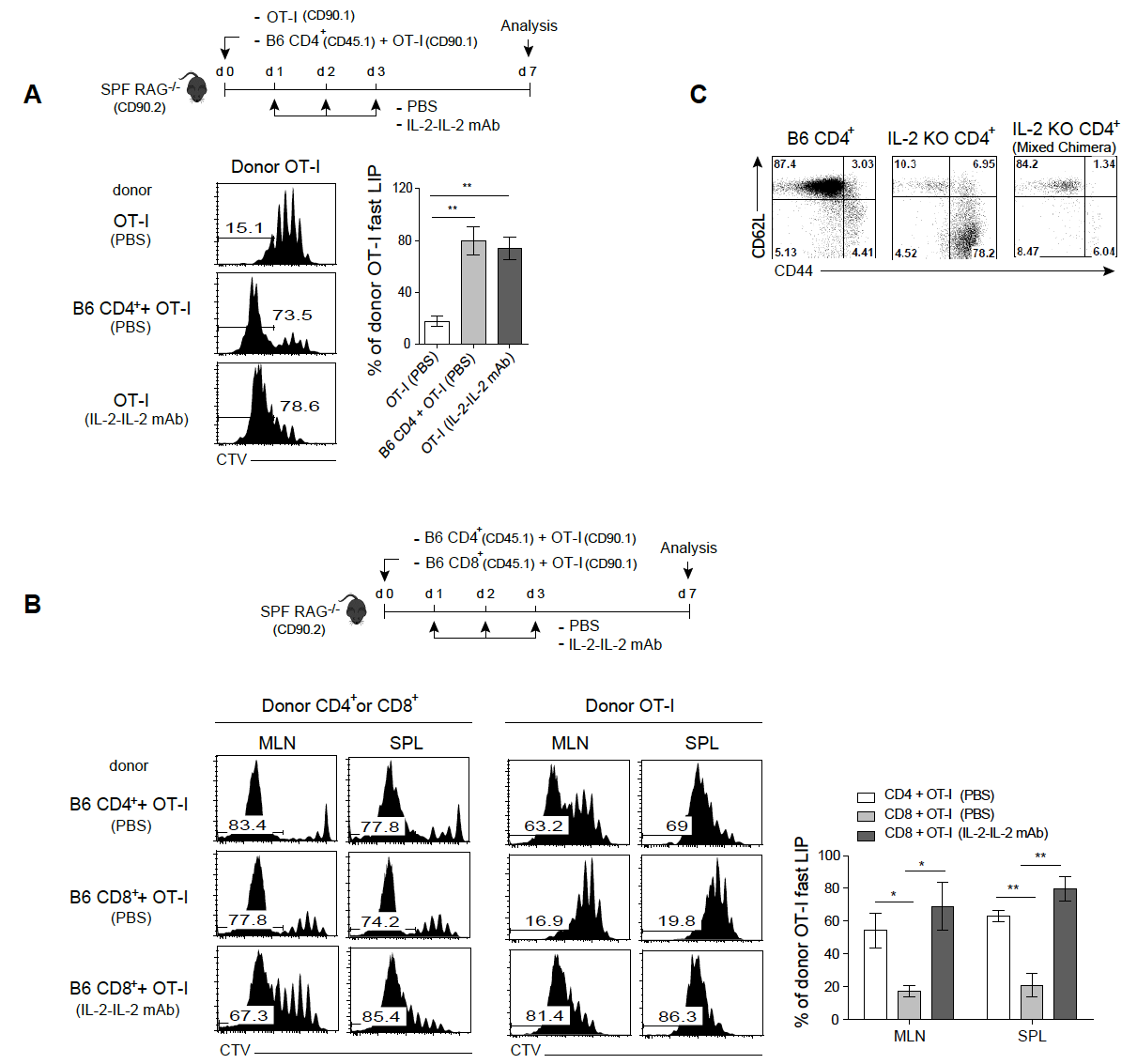
**

**Figure S6**. **The slow rate of LIP is augmented by enhanced levels of IL-2 to show the fast and robust response. (A)** CTV-labeled OT-I CD8^+^ T cells (CD90.1; 5 × 10^5^ cells) either alone or along with polyclonal naïve CD4^+^ T cells (CD45.1; 1 × 10^6^ cells) were injected i.v. into SPF RAG^−/−^ hosts (CD90.2; top). The mice were then injected i.p. either with PBS or with IL-2/anti-IL-2 mAb complexes at the indicated time points (top). CTV dilution (bottom left) and percentage of the fast LIP of donor OT-I cells (bottom right) were analyzed on day 7 by flow cytometry. **(B)** A mixture of CTV-labeled OT-I CD8^+^ T cells (CD90.1; 5 × 10^5^ cells) and either polyclonal naïve CD4^+^ (CD45.1; 1 × 10^6^ cells) or polyclonal naïve CD8^+^ T cells (CD45.1; 1 × 10^6^ cells) was injected i.v. into SPF RAG^−/−^ hosts (CD90.2; top). The mice were then injected i.p. either with PBS or with IL-2/anti-IL-2 mAb complexes at the indicated time points (top). CTV dilution (bottom left two panels) and percentages of the fast LIP of donor OT-I cells (bottom right) were analyzed on day 7 by flow cytometry. **(C)** Bone marrow (BM) cells from B6 mice (CD45.1; 2 × 10^6^ cells) were mixed with BM cells from IL-2 KO mice (CD90.2; 2 × 10^6^ cells) at a 50:50 ratio and then injected i.v. into lethally irradiated (900 cGy) B6.PL mice (CD90.1). At 8 weeks after BM transfer, the resulting chimeric mice were analyzed by flow cytometry for the expression of CD44 and CD62L gated on CD4^+^ T cells. Data shown in **(A)** and **(B)** are the mean ± SEM (*n* = 3-4 mice per group) and are representative of at least three independent experiments. **p* < 0.05; ***p* < 0.01.

**Figure S7**


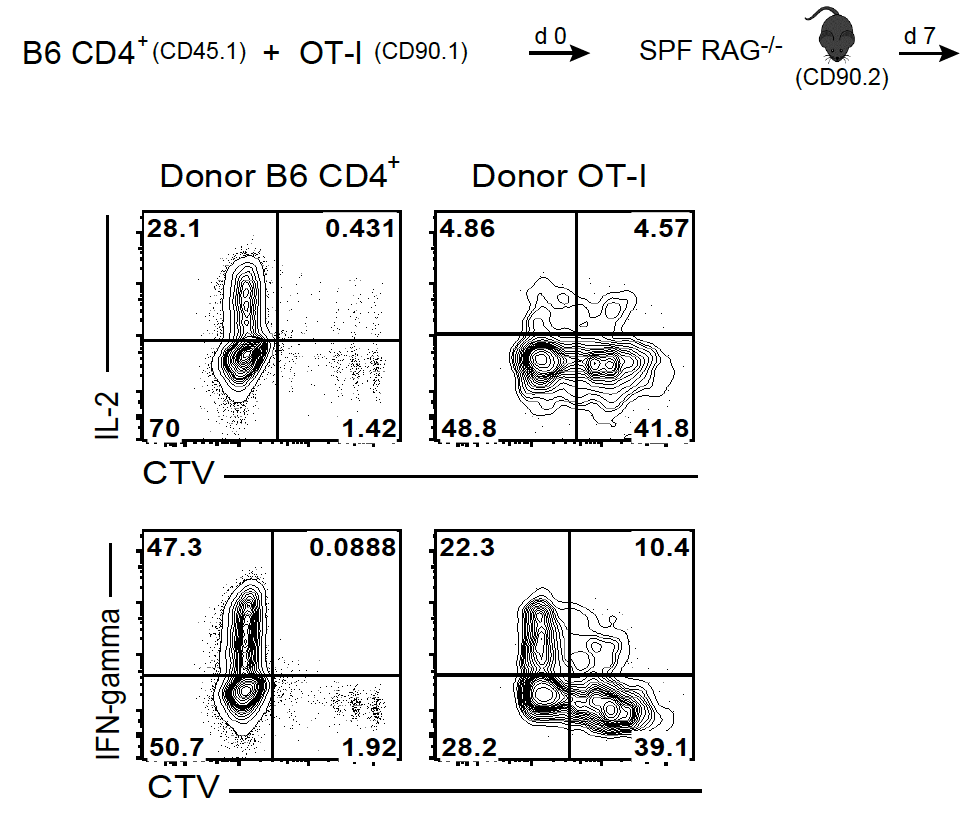


**Figure S7**. **Production of IL-2 and IFN-γ from donor T cells in SPF RAG^−/−^ hosts.** CTV-labeled polyclonal naïve CD4^+^ T cells (CD45.1; 1 × 10^6^ cells) and OT-I CD8^+^ T cells (CD90.1; 5 × 10^5^ cells) were i.v. injected into SPF RAG^−/−^ hosts (CD90.2; top). At day 7, the mice were then analyzed on by flow cytometry for intracellular IL-2 and IFN-γ synthesis after 4 hr *in vitro* restimulation with PMA and ionomycin (bottom).

**Figure S8**

**
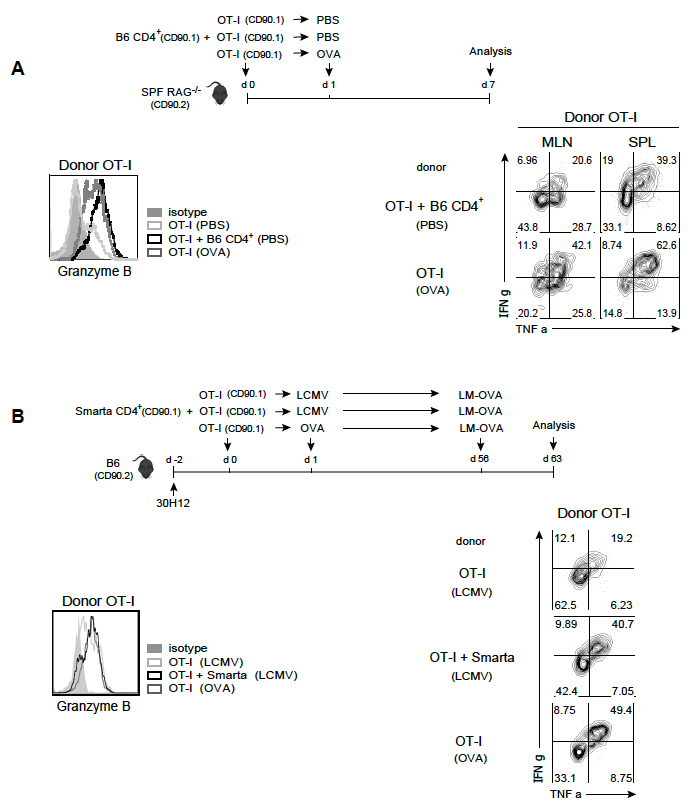
**

**Figure S8**. **The LIP response driven by IL-2 leads to the differentiation into functional effector and memory cells. (A)** OT-I CD8^+^ T cells (CD90.1; 5 × 10^5^ cells) were injected i.v. either alone or along with polyclonal naïve CD4^+^ T cells (CD90.1 or CD45.1; 1 × 10^6^ cells) into SPF RAG^−/−^ hosts (CD90.2) and then were either left with PBS injection or immunized *i.p.* with OVA protein (100 µg/mouse; top). The mice were then analyzed on day 7 by flow cytometry for the expression of granzyme B (bottom left; SPL) and intracellular staining of IFN-γ and TNF-α (bottom right; MLN and SPL) gated on donor OT-I cells. Data are representative of at least three independent experiments (*n* = 4-6 mice per group). **(B)** B6 mice (CD90.2) were treated i.p. with anti-Thy1.2 mAb (30H12) 2 days before cell transfer and then injected i.v. with OT-I CD8^+^ T cells (CD90.1; 5 × 10^5^ cells) either alone or along with SMARTA CD4^+^ T cells (CD90.1; 5 × 10^4^ cells) and l day later, immunized i.p. either with LCMV Armstrong (2 × 10^5^ PFU) or with OVA protein (100 µg/mouse; top). At 56 days after adoptive transfer, the mice were then challenged with OVA-expressing *Listeria monocytogenes* (LM-OVA) via oral gavage (5 × 10^10^ CFU; top). The mice were analyzed on day 7 post-challenge for granzyme B (bottom left; SPL) and intracellular staining of IFN-γ and TNF-α (bottom right; MLN and SPL) of donor OT-I cells. Data are representative of at least three independent experiments (*n* = 5 mice per group).
